# Supplementary figures and images for: Differential assembly of RNP granules via activation of distinct dsRNA sensors by adenovirus mutants
Source: PLoS Pathog. 2026 Jul 24;22(7):e1014452. doi: 10.1371/journal.ppat.1014452 (PMC13426940; doi:10.1371/journal.ppat.1014452)

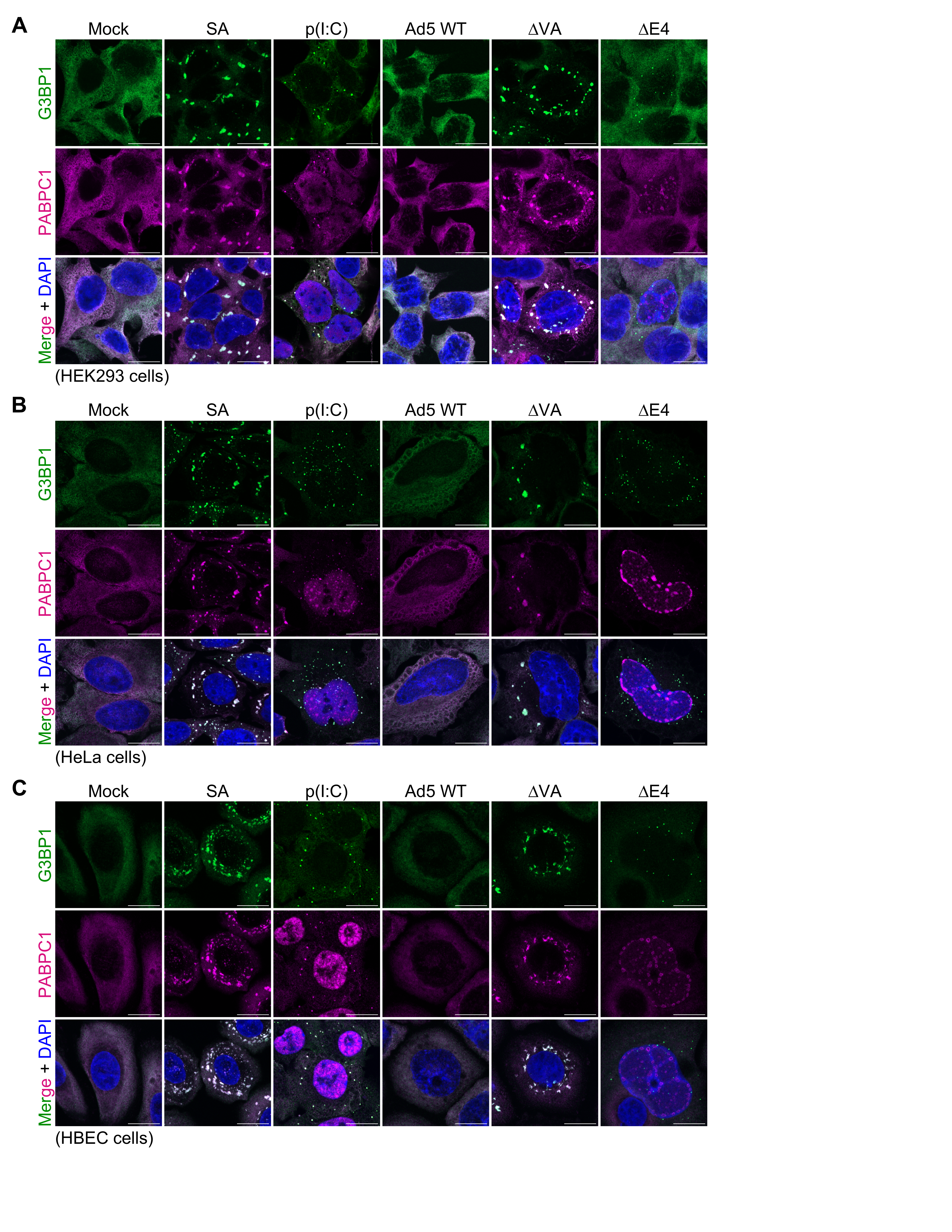

Supplement: S1 Fig — Cell lines (A) HEK293, (B) HeLa and (C) HBEC were infected with Ad5 WT, ∆VA or ∆E4 at an MOI of 10, or treated with sodium arsenite (SA, 0.5 mM for 1 h) and poly(I:C) (0.5 µg/mL for 6 h), as indicated. Infected cells were fixed at 24 hpi for Ad5 WT and ∆VA, or at 40 hpi for ∆E4. Cells were stained for the granule markers G3BP1 (green) and PABPC1 (magenta), and nuclei is marked with DAPI (blue). SGs were observed in SA-treated and ∆VA-infected cells. RLBs were observed in poly(I:C)-treated and ∆E4-infected cells. No granules were observed in untreated or WT-infected cells. Scale bar = 15 µm. (TIF) [file ppat.1014452.s001.TIF]

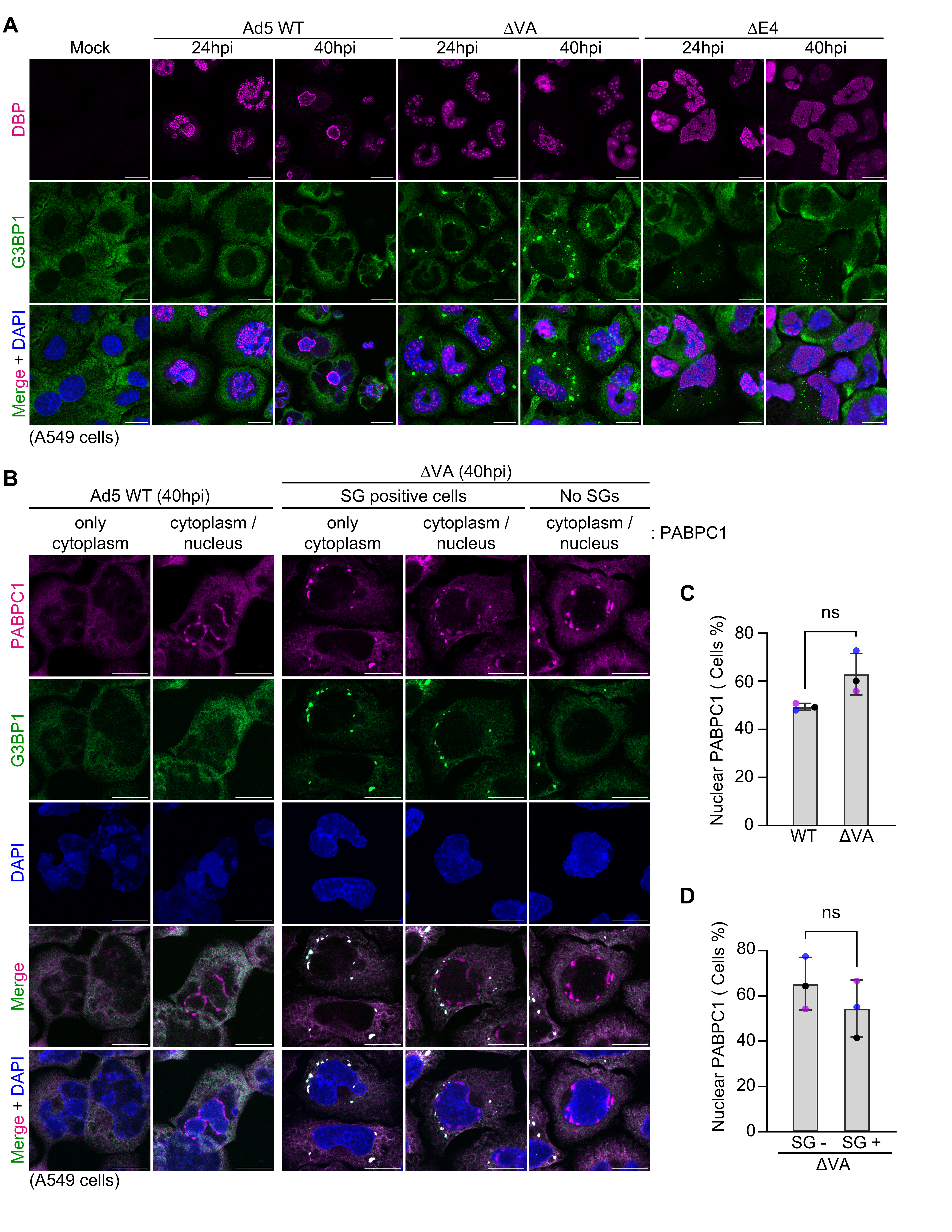

Supplement: S2 Fig — (A) A549 cells were uninfected or infected with Ad5 WT, ΔVA, or ΔE4 at MOI 10 and fixed at 24 or 40 hpi. Cells were stained for the viral protein DBP (magenta), G3BP1 (green), and DAPI (blue). (B) Representative fields of Ad5 WT and ∆VA-infected A549 cells at 40 hpi co-stained for G3BP1 (green) and PABPC1 (magenta). Images show cells in which PABPC1 is either detected exclusively in the cytoplasm or exhibits a distinct nuclear localization. (C) The proportion of cells showing nuclear PABPC1 signal was quantified for both WT and ΔVA infections. (D) Nuclear PABPC1 was also quantified in ∆VA-infected cells which were either positive or negative for stress granules, showing similar proportions between both groups. Bars represent mean and error bars indicate standard deviation, with paired replicate values indicated by colored dots. Statistical significance was assessed using two-tailed paired t-test, with ns = not significant. Scale bar = 15 µm. (TIF) [file ppat.1014452.s002.TIF]

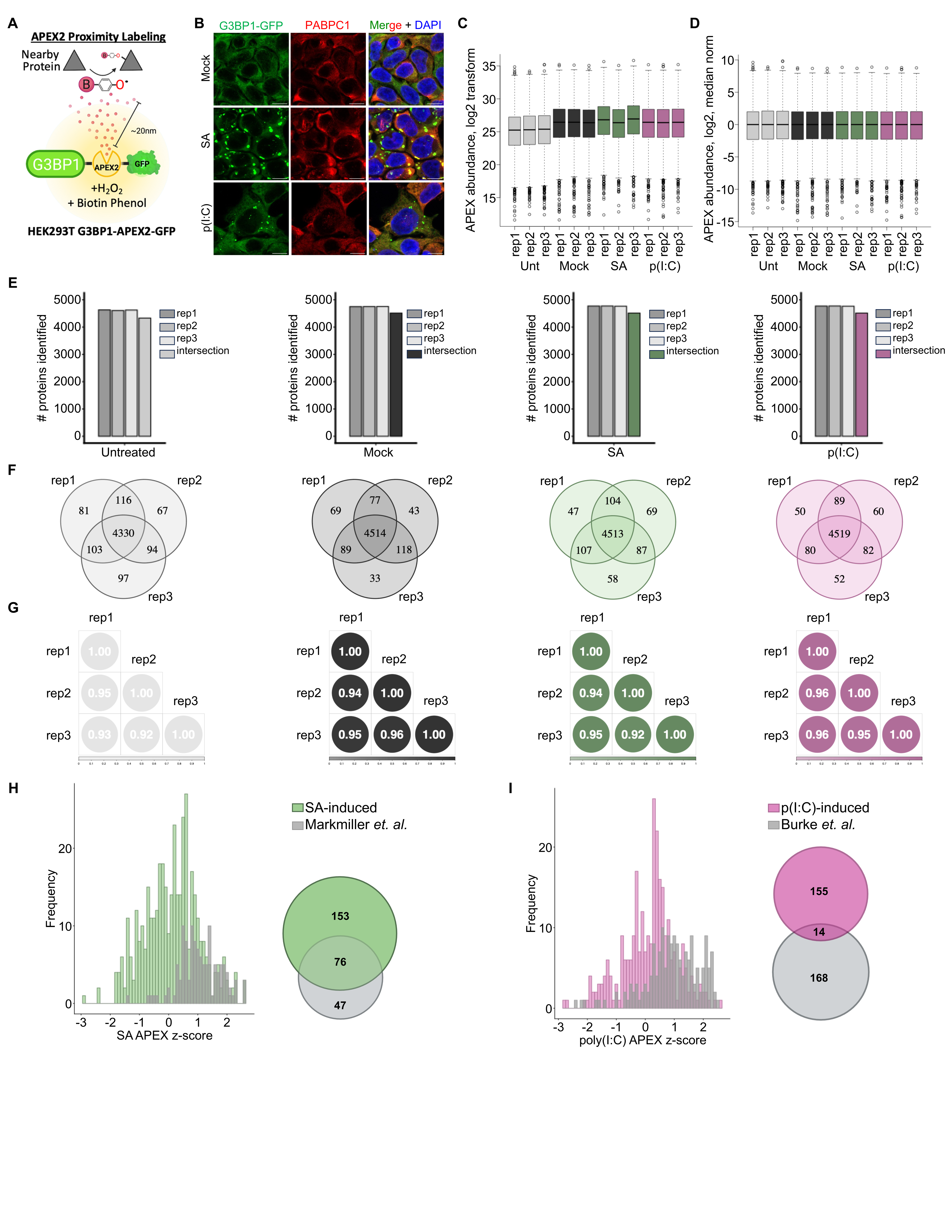

Supplement: S3 Fig — (A) Schematic representation of APEX2 proximity labeling, indicating selective targeting of proteins in close proximity to G3BP1 (~20 nm radius). (B) Immunofluorescence in HEK293T cells expressing G3BP1-APEX2-GFP following treatment with sodium arsenite (SA, 1.0 mM for 1 h) or poly(I:C) (1.0 µg/mL for 4 h). Cells were co-stained for PABPC1 (red) and nuclei (DAPI). Scale bar = 15 µm. (C-G) Data normalization and reproducibility of APEX2 proteomic datasets across untreated (light grey), mock (dark grey), SA (green), and poly(I:C) (magenta) treatments for each replicate. (C,D) Boxplots showing log2 transformed (C) and median normalized APEX abundance data (D) for each replicate. (E) Bar charts showing the number of APEX proteins identified for each replicate and their intersection across replicates. (F) Venn diagrams showing the overlap of APEX proteins identified between replicates. (G) Correlation plots showing correlation coefficients for APEX abundance comparions across replicates. Correlation plots were generated using the corrplot package in R (version 0.95 built in R version 4.4.1) with correlations calculated for pairwise complete observations. (H) Distribution of SA APEX z-scores for all proteins identified by G3BP1-APEX proximity labeling during SA treatment (green) and proteins identified by Markmiller et al. (grey). The Venn diagram shows the intersection of predicted SA-induced stress granule proteins in our dataset (defined by SA z-score > 0) and stress granule proteins identified by Markmiller et al. The z-score threshold was selected based on the overlap between the two datasets. (I) Distribution of poly(I:C) APEX z-scores for all proteins identified by G3BP1-APEX proximity labeling during poly(I:C) treatment (magenta) and proteins identified by Burke et al. (grey). Venn diagram showing intersection of predicted poly(I:C)-induced RLB proteins in our dataset (defined by poly(I:C) z-score > 0) with proteins identified in Burke et al. The z-score threshold [file ppat.1014452.s003.TIF]

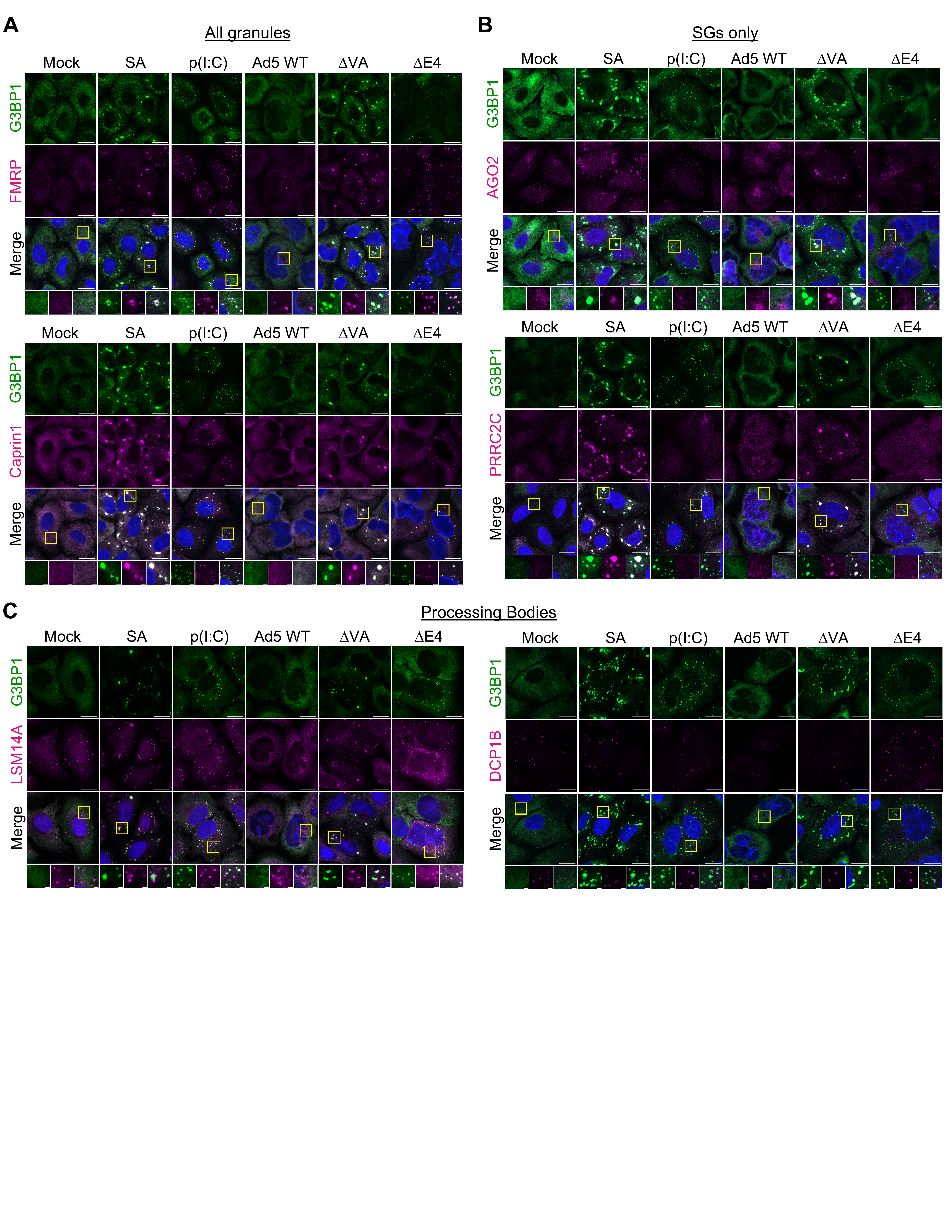

Supplement: S4 Fig — (A-C) A549 cells were infected with WT Ad5, ∆VA and ∆E4 MOI 10 for 40 h or treated with sodium arsenite (SA, 0.5 mM 1 h) and poly(I:C) (0.5 μg/mL for 6 h) as indicated. Panels show validation of RNA binding proteins identified by APEX analysis as being differentially recruited to SGs and RLBs. G3BP1 staining (green) was used as a marker for granule assembly, and nuclei are stained with DAPI (blue). (A) Staining for FMRP and Caprin1 (magenta) show the recruitment of proteins to both granules. (B) Staining for PRRC2C and AGO2 (magenta) indicates differential granule composition, with proteins present only in SGs induced by sodium arsenite or ∆VA infection and absent from RLBs. (C) Co-staining of G3BP1 with P-body proteins LSM14A and DCP1B demonstrates close proximity between SGs/RLBs and P-bodies, with partial recruitment of LSM14A to both types of granules. Scale bar = 15 µm (main panels), 2 µm (cropped panels). (TIF) [file ppat.1014452.s004.TIF]

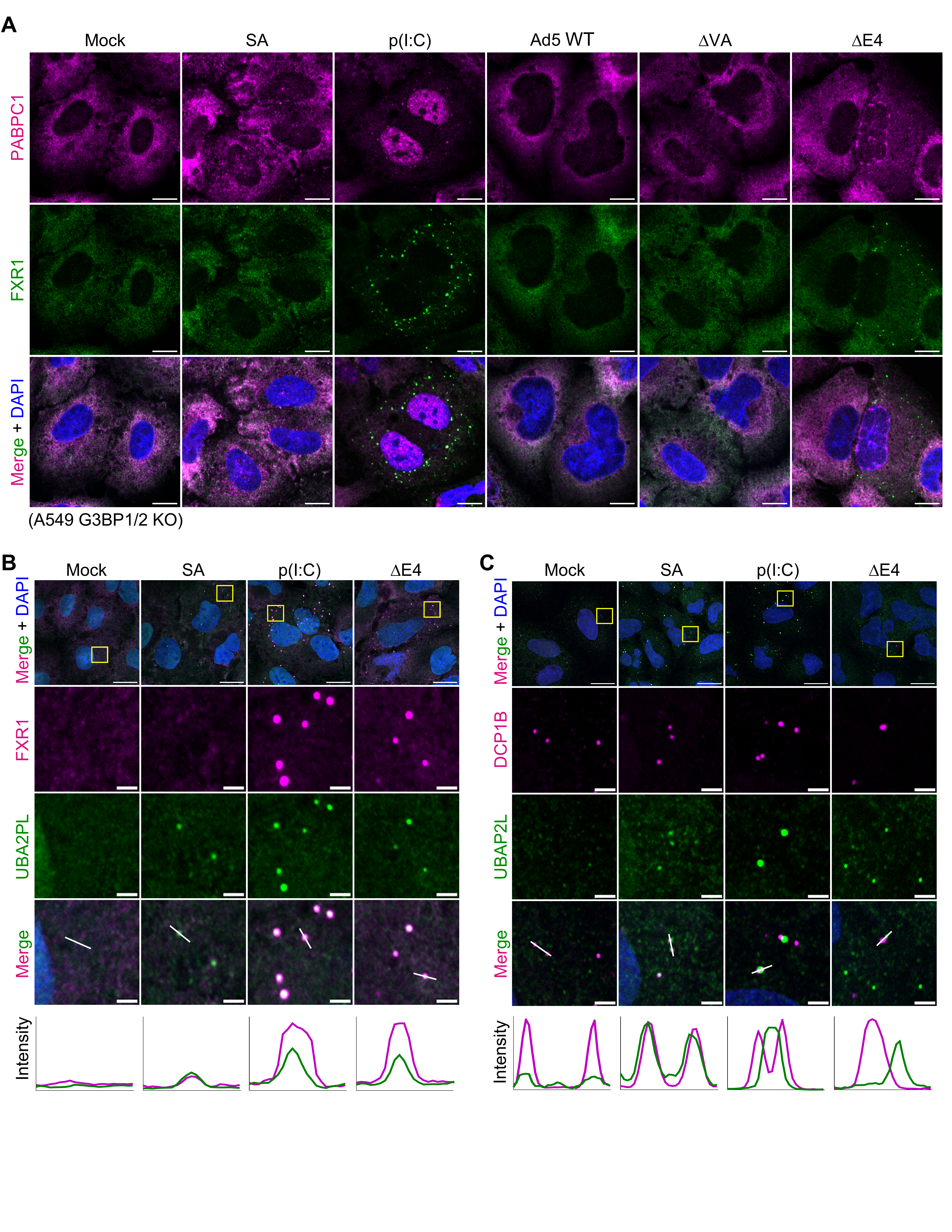

Supplement: S5 Fig — (A) A549 cells lacking expression of G3BP1 and G3BP2 (G3BP1/2 KO) were infected with Ad5 WT, ∆VA and ∆E4 MOI 10 for 24 h, or treated with sodium arsenite (SA, 1.0 mM 1 h) and poly(I:C) (0.5 μg/mL for 6 h), as indicated. Cells were stained for PABPC1 (magenta) and FXR1 (green), showing RLB assembly in response to poly(I:C) and ∆E4 infection. (B) Alternatively, G3BP1/2 KO cells were treated with SA, poly(I:C) or infected with ∆E4 and stained for FXR1 (magenta) and UBAP2L (green). Cropped panels and fluorescence intensity profiles show co-enrichment of FXR1 and UBAP2L in RLBs induced by poly(I:C) and ∆E4. (C) By contrast, co-staining for DCP1B (magenta) and UBAP2L (green) and analysis by fluorescence intensity profiles show that RLBs are distinct from P-bodies, whereas SA treatment promotes recruitment of UBAP2L to P-bodies stained by DCP1B. White bars indicate the regions used for fluorescence intensity profiling. Nuclei were stained with DAPI (blue). Scale bar = 15 µm (main panels), 2 µm (cropped panels). (TIF) [file ppat.1014452.s005.TIF]

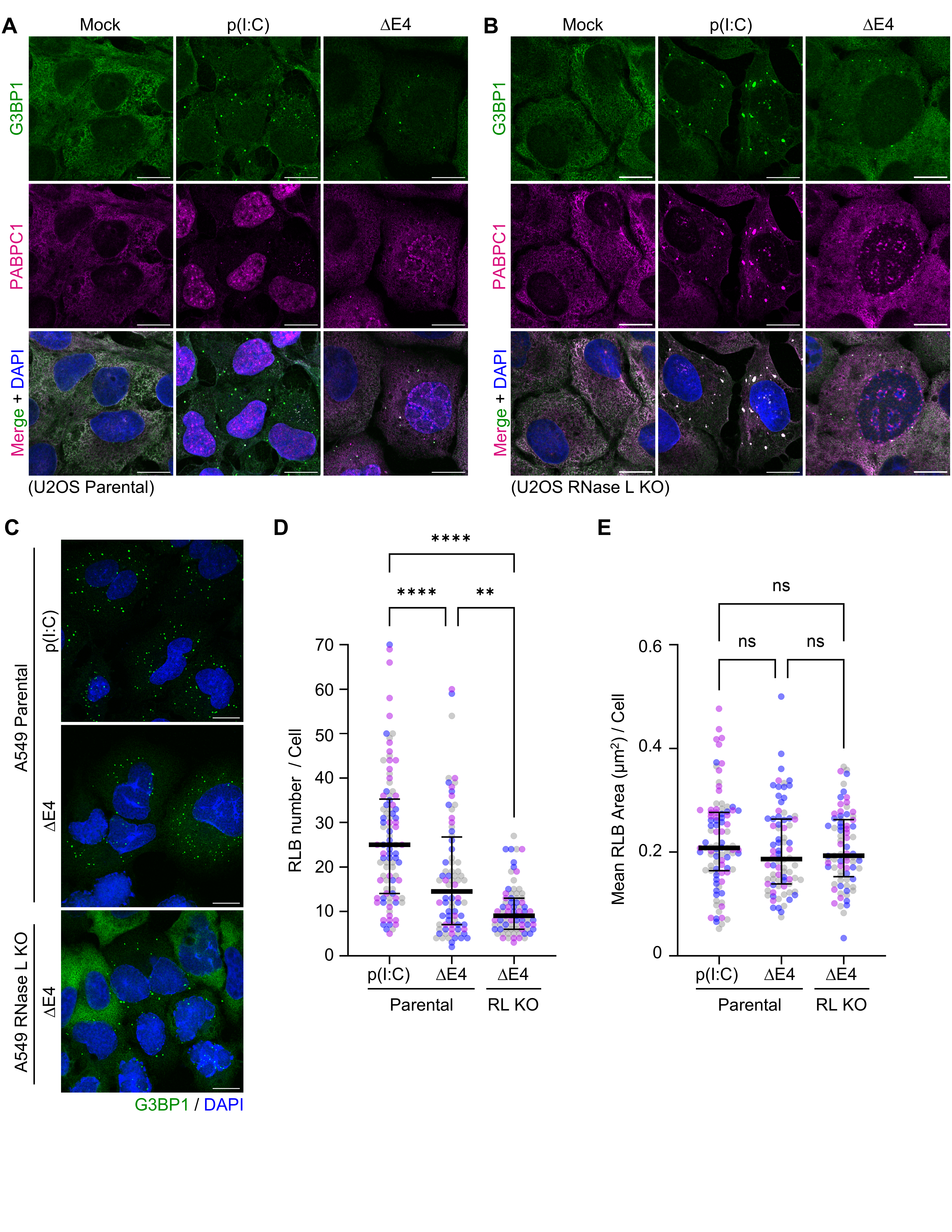

Supplement: S6 Fig — (A) U2OS parental and RNase L KO cells (B) were treated with poly(I:C) (1 µg/mL for 6 h) or infected with ∆E4 at MOI 10 for 40 h. Cells were stained for G3BP1 (green) and PABPC1 (magenta), and with DAPI for nuclei (blue). Poly(I:C) induced RLB and stress granule assembly in parental and RNase L KO cells, respectively. In contrast, ∆E4 infection induced RNP granules resembling RLBs in both cell lines. (C-E) A549 parental or RNase L KO cells were treated with poly(I:C) or infected with ΔE4 as above and stained for G3BP1 (green) and DAPI (blue). Representative fields are shown in (C). Number of granules per cell (D) and mean granule area (µm2) per cell (E) were quantified for each condition. The total number of cells analyzed across biological replicates was poly(I:C) n = 94, ∆E4 in parental cells n = 80, ∆E4 in RNase L KO n = 78. Individual cell values are shown with dots in the graphs, with colors indicating biological replicates. Black bars indicate median and interquartile range. Statistical significance was assessed by Kruskal-Wallis test followed by Dunn’s multiple comparisons test. ns = not significant, ** = P < 0.01, **** = P < 0.0001. Scale bar = 15 µm. (TIF) [file ppat.1014452.s006.TIF]

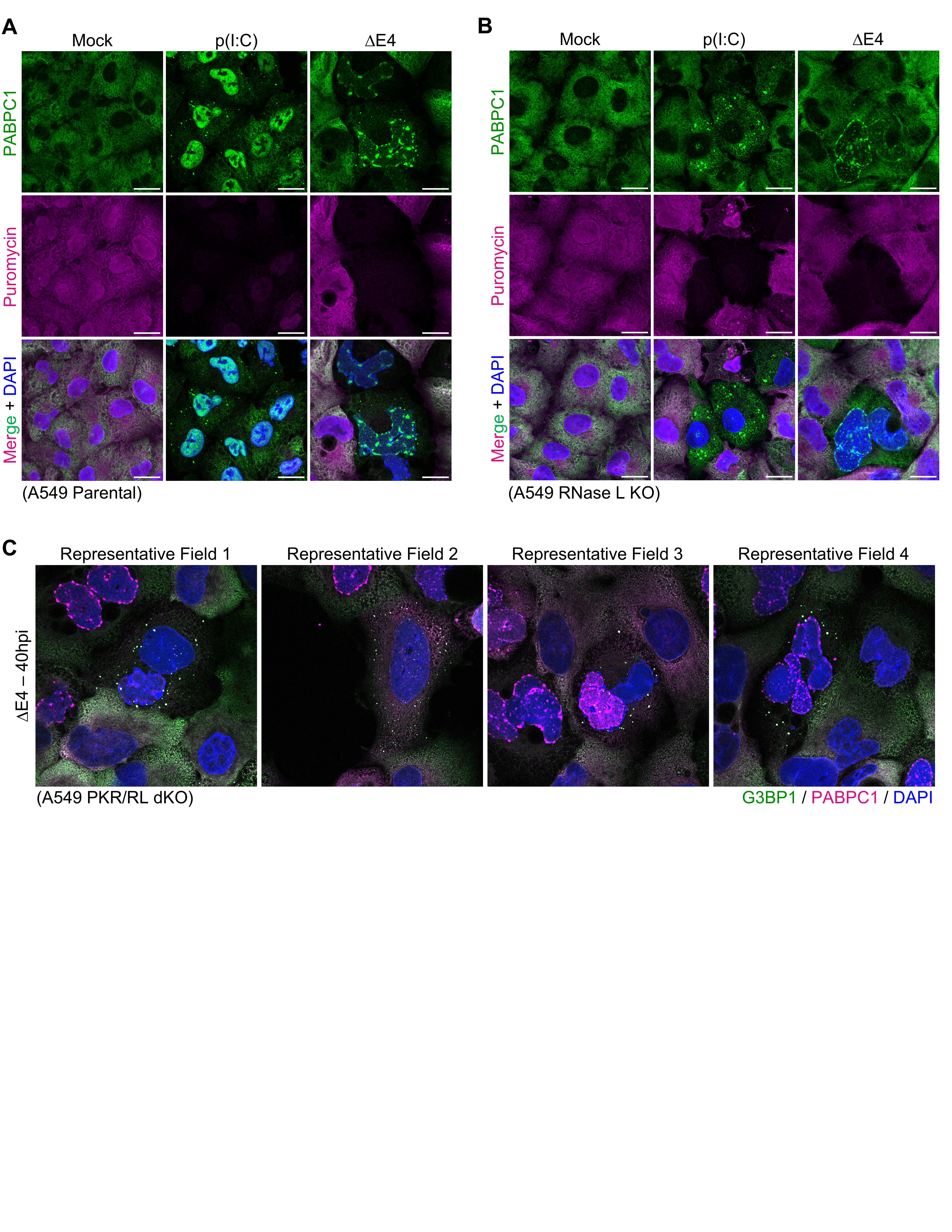

Supplement: S7 Fig — (A) A549 parental and RNase L KO cells (B) were treated with poly(I:C) (1 µg/mL for 6 h) or infected with ∆E4 (MOI 10 for 40 h). Before fixation, cells were treated with puromycin (10 µg/mL) for 10 min to measure translation. Cells were stained for puromycin incorporation (magenta) and G3BP1 (green). (C) Additional representative fields of ΔE4-infected A549 PKR/RNase L double-KO cells at 40 hpi stained for G3BP1 (green) and PABPC1 (magenta). Images show that cytoplasmic G3BP1/PABPC1-positive RLB-like granules can still be detected in cells deficient for PKR and RNase L expression. Nuclei were stained with DAPI (blue). Scale bar = 15 µm. (TIF) [file ppat.1014452.s007.TIF]

Figure 1B

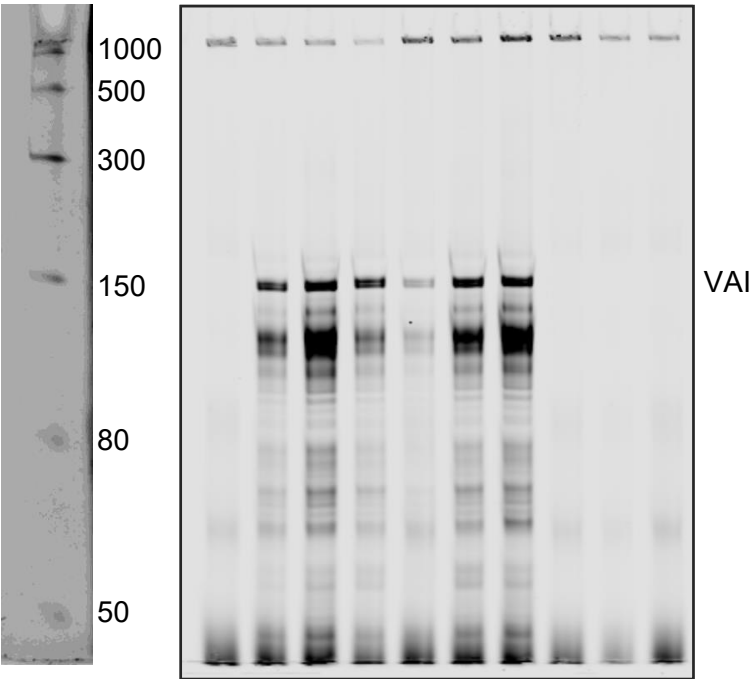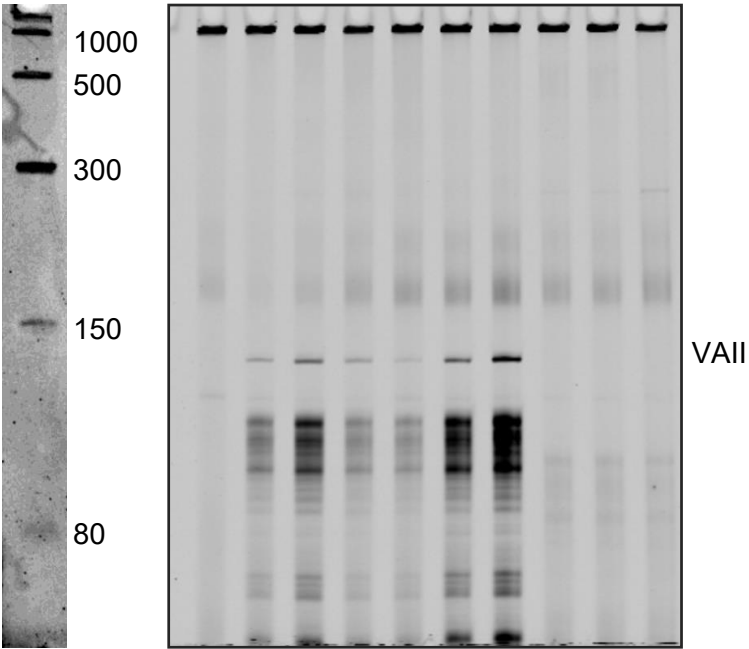

Low Range ssRNA Ladder (NEB, N0364S)

Figure 1C

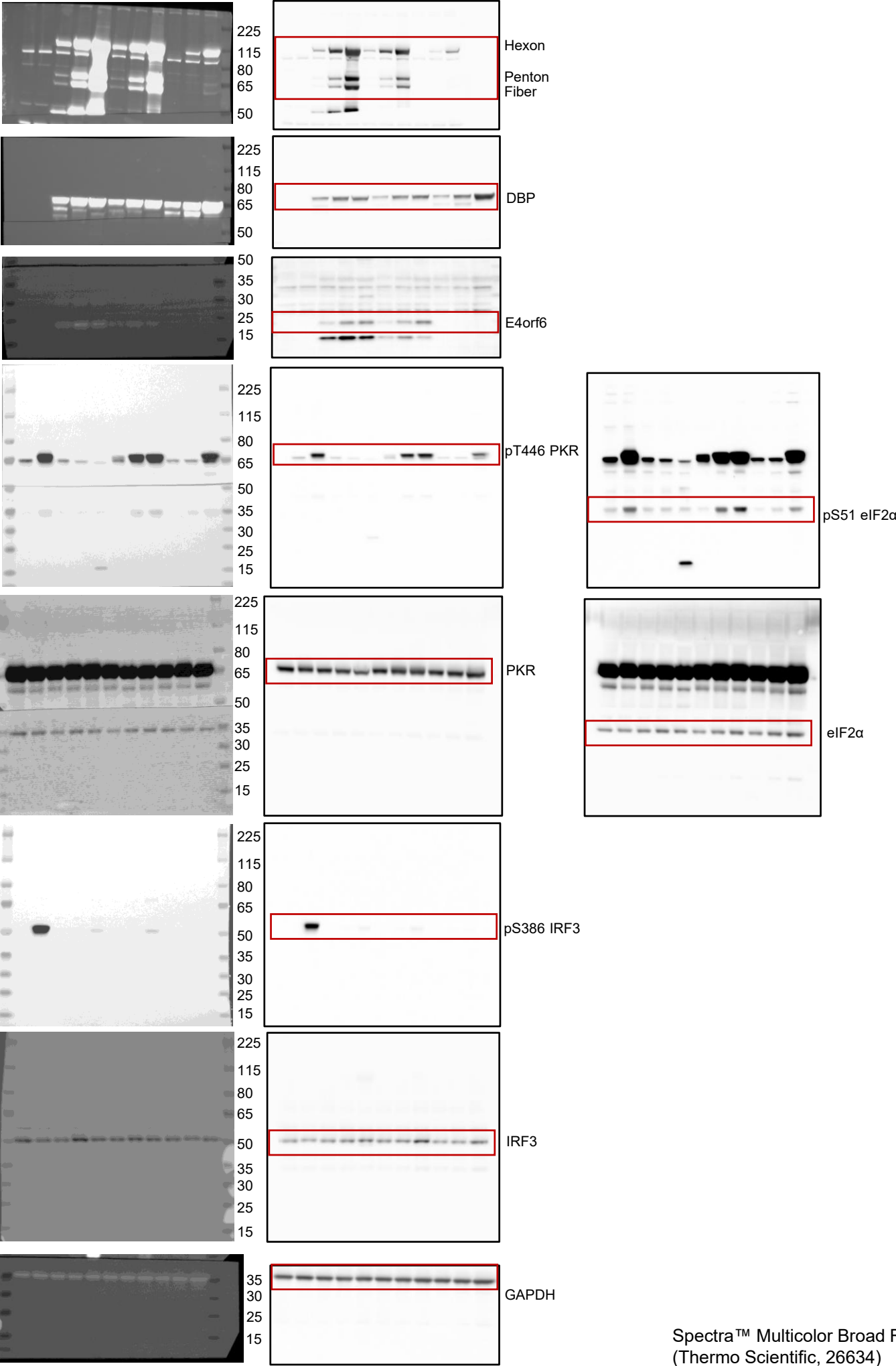

Figure 4A

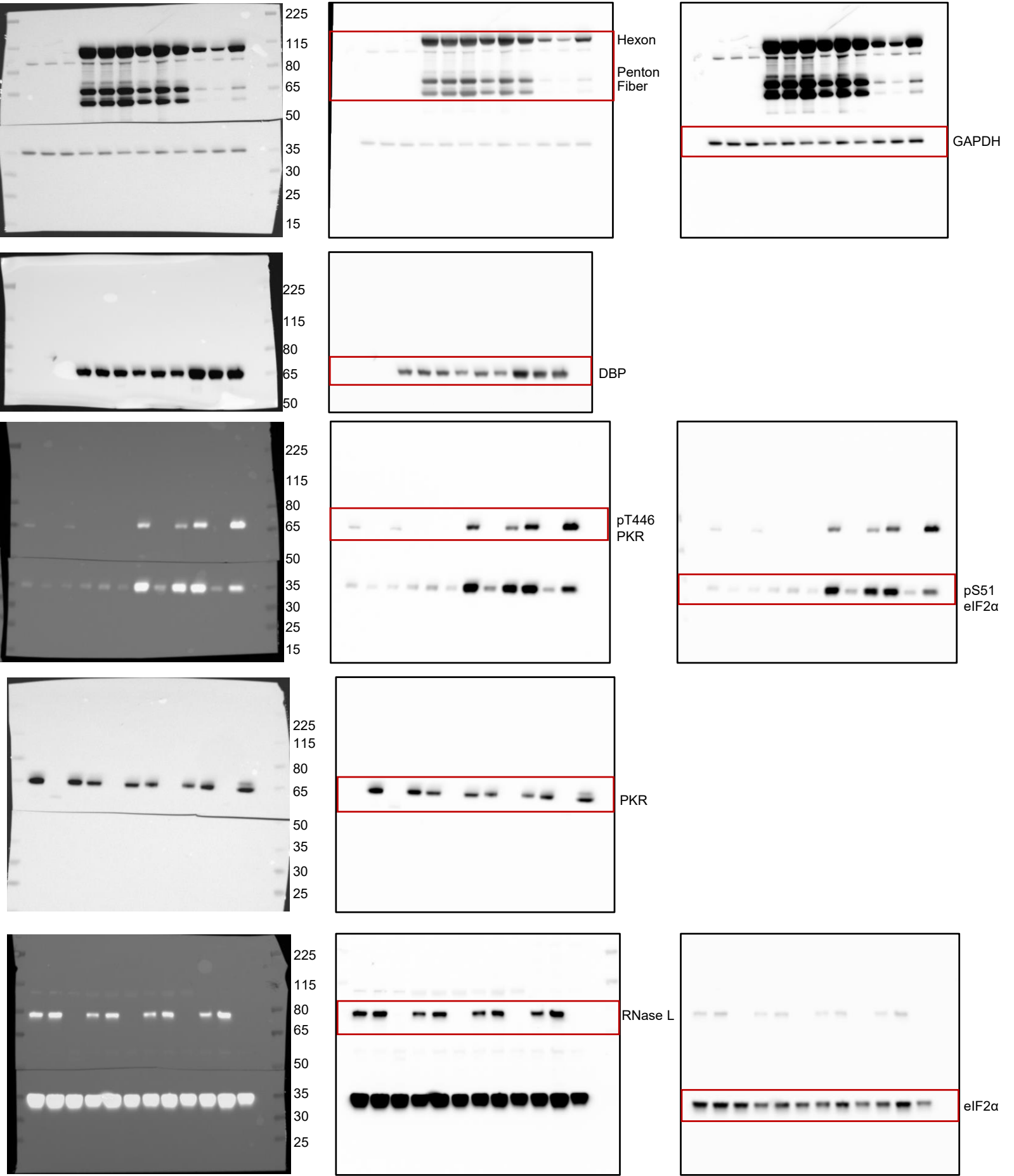

Figure 7C

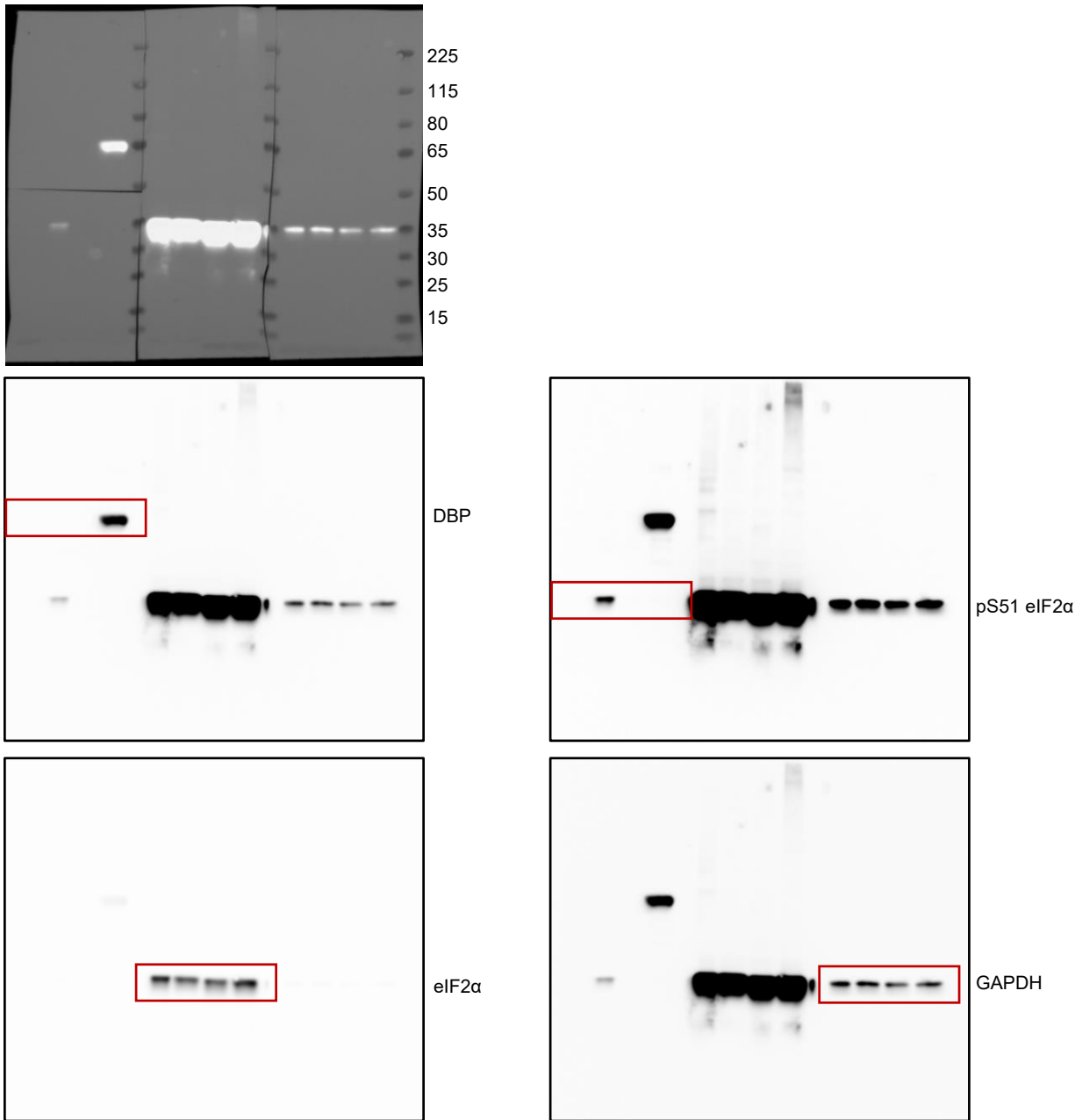

Supplement: S1 File — (PDF) [file ppat.1014452.s009.pdf]
