# Supplementary material for: Differential assembly of RNP granules via activation of distinct dsRNA sensors by adenovirus mutants
Source: PLoS Pathog. 2026 Jul 24;22(7):e1014452. doi: 10.1371/journal.ppat.1014452 (PMC13426940; doi:10.1371/journal.ppat.1014452)
Supplement: Attachment — Submitted filename: Response to Reviewers.docx [file ppat.1014452.s012.docx]

**Reviewer #1**

**Part I – Summary**

In this manuscript, Steinbock et al demonstrate how different adenovirus (Ad5) mutants induce pathways that lead to different molecular aggregate formation, including PKR-dependent stress granules (SGs) and RNAseL-dependent bodies (RLBs). Although a very thorough study, I struggled to grasp the novelty since most of the data included is confirming the structural properties of the aggregates that have been previously described (mostly by the Parker lab), only this time using Ad5 to induce them. I am also not convinced that the granules induced by the ΔE4 mutant are distinct from RLBs, especially because the images selected to support this claim are not convincing. Additionally, some of the figures seem to be repetitive, demonstrating the same conclusion in different ways. I recommend merging some of these figures or changing the order for a more cohesive story. I also recommend shifting the rhetoric (especially in the discussion) to focus on how these pathways are being inhibited by the WT virus and how the effect of having these aggregates formed in the mutants affect the outcome of the infection. That way, you can focus on the importance for the virus to block these pathways, rather than focusing on phenomena that doesn’t even occur in the WT virus. This shift can also reduce the “me too” feel of the study.

R: We appreciate the reviewer’s careful evaluation of the manuscript. We have revised the manuscript to address comments and suggestions, and to clarify better the novelty of our study.

While several structural features of SGs and RLBs have been described previously, our study uses Ad5 mutants to show how distinct viral defects activate different dsRNA-sensing pathways during infection, leading to PKR-dependent SGs during ΔVA infection and RLB-like granules during ΔE4 infection. We also reframed our claims to clarify that ΔE4-induced granules persist in RNase L KO and PKR/RNase L double-KO cells while still acknowledging that RNase L activity may contribute to overall granule assembly in parental infected cells. Together, these observations offer new evidence for additional mechanisms of granule assembly upon viral infection and/or nuclear dsRNA accumulation.

We also revised the text to make the narrative clearer by emphasizing that activation of dsRNA-sensing pathways occurs in the context of specific defects caused by the viral mutations. In brief, VA RNAs normally inhibit PKR activation, whereas E4 proteins counteract host proteins that restrict viral genome replication and viral RNA processing. Therefore, loss of these viral elements leads to distinct primary defects, including PKR-dependent translational arrest during ΔVA infection and defective processing of viral late transcripts during ΔE4 infection, which subsequently promote granule assembly. Finally, we have modified and included several figures and supplementary panels to improve narrative flow, reduce redundancy, and include additional quantification and representative images supporting the revised conclusions.

**Part II – Major Issues**

Figure 1C. Your ΔE4 mutant virus has drastically lower levels of viral proteins. Do these viruses replicate similarly? If not, is it even fair to compare activation of sensors and pathways? I would like to see viral replication kinetic data to confirm these mutant viruses are not highly attenuated compared to the WT.

R: We agree with the reviewer that differences in viral replication could introduce caveats when comparing phenotypes induced by viral mutants.

As previously described in detail, the ΔE4 mutant virus is highly attenuated in comparison to wild-type Ad5. The ΔE4 mutant lacks E4 early proteins, including E4orf3 and E4orf6, which are necessary for counteracting host defenses. Our lab has previously characterized several host proteins involved with viral restriction which are antagonized by E4 proteins, such as the MRE11-RAD50-NBS1 complex, TFII-I and hnRNPC/RALY [1–4]. The expression of E4orf6 is especially important for proper splicing of late genes [4]. Mutants lacking this gene, including the ΔE4 mutant used in this work, are splicing-defective and produce viral dsRNA, as previously described by Price *et al.*, 2022 [5].

To compare viruses, titers were normalized by PFU number in permissive W162 cells, a Vero-derived cell line which expresses the adenovirus E4 coding region to facilitate complementation of defective mutants [6]. In this cell line viral infection leads to activation of E4 proteins in response to the viral E1A protein, rescuing the defects in viral splicing and replication [7]. Thus, viruses were compared using matched infectious input, but they are not expected to replicate similarly in A549 cells. In A549 cells, expression of late proteins is strongly attenuated but comparable levels of early protein expression are achieved (e.g. DBP expression, as shown in **Fig 1C**). We have clarified this point in the revised text mentioning the replication defect of this mutant to avoid the interpretation as a direct comparison between viruses with equivalent replication kinetics (**Lines 158-162**).

Figure 2A. Are these aggregates forming specifically in infected cells or bystander cells? Could the induction be occurring in a viral-RNA independent matter? There is evidence that host dsRNA can induce PKR activation (Kim et al 2018, Molecular Cell). I would like to see aggregate + viral protein staining to determine if the pathway induction is dependent on the virus being inside of the cell or is it through a paracrine manner.

R: We agree with the reviewer that it is important to distinguish granule formation in infected cells from bystander cells. To address this point, we included co-staining of G3BP1 and the viral protein DBP to demonstrate that granule assembly occurs in infected cells (**S2 Fig A**). These images confirm that cytoplasmic granules are present in DBP-positive cells, supporting our conclusions that granule assembly is a downstream response to viral mutant infection rather than induction through a paracrine mechanism.

Since RLB assembly accompanies PABPC1 nuclear import, co-staining of G3BP1 and PABPC1 was used in the main figures to facilitate distinction between granules. Since infections were performed at high MOIs (10 PFU / cell), most cells were expected to be infected. In infected cells, cytopathic effect was also visualized by changes in cell morphology and nuclear enlargement, as previously reported by Avgousti *et al.*, 2016 and Charman *et al*., 2023 [8,9]. Consistent with this, granule assembly followed the progression of infection, as shown by time-course immunoblotting (**Fig 1C**) and granule quantification over time (**Fig 2A-C**).

General comment: All infections with KO cells should be accompanied with infection kinetics (titers or qPCR) to ensure that the differences observed are not due to differences in viral replication. Are the granules not present because the protein is KO or because the virus is not replicating at all in these cells? Or is the virus replication 10 times more in these cells? WB data is not very quantitative and not very informative when it comes to comparing “infections levels”.

R: We agree with the reviewer that differences in viral replication should be considered when interpreting granule assembly in KO cell lines.

Infection with the ΔVA mutant is restricted by PKR-dependent translational arrest. It has been reported that defects in protein expression are partially rescued in cells lacking PKR expression [5,10]. In this sense, ΔVA replication is expected to be higher in PKR KO cells, but stress granule assembly will not be observed since this phenotype is dependent on PKR activation.

By contrast, the ΔE4 mutant is highly attenuated as viral genomes and viral transcripts are targeted by different host proteins in the absence of the viral proteins E4orf3 and E4orf6, strongly impacting viral genome replication and late protein expression, as mentioned above [1–4].

To address this concern, we repeated experiments using independently generated and validated A549 PKR KO and RNase L KO clones. Since immunoblotting is not ideal for quantitative comparison of infection levels, we have also included qPCR analysis of viral genome accumulation under the same experimental conditions. Immunoblotting showed a modest increase in viral late protein expression during ΔVA infection in PKR KO cells, consistent with partial rescue of the ΔVA translational defect, but not in RNase L KO cells (**Fig 4A**). More importantly, qPCR showed a small but significant increase in ΔVA viral genome accumulation in PKR KO cells, but not in RNase L KO cells (**Fig 4B**). Comparatively, we did not observe rescued late protein expression or viral genome accumulation during ΔE4 infection in either PKR KO or RNase L KO cells, in agreement with prior literature.

**Part III – Minor Issues**

Line 119: It is unclear what the ΔE4 mutant virus is. What is E4 and why does the virus produce dsRNA in its absence? I recommend adding more background on this mutant to make sense of why it is being used and why it produces dsRNA. This is only briefly mentioned in the results.

R: The background for E4-deletions was included in the Introduction (**lines 100-109**), where we explain that AdV transcription from both DNA strands can generate viral dsRNA and that efficient viral pre-mRNA processing depends on the E1B-55K/E4orf6 complex. We also note that splicing-defective mutants lacking these proteins accumulate nuclear dsRNA and activate PKR. This point is revisited in the Discussion (**lines 391-393**). To make the Results section clearer, we have revised the first mention of ΔE4 to define it explicitly as an E4-deleted, splicing-defective mutant that accumulates viral dsRNA due to impaired viral RNA processing (**lines 141-144**).

Figure 2B. The figure legend should clarify how many cells were counted. 4 out of 10 cells and 80 out of 200 is still 40%, but it doesn’t mean the same in terms of significance. The figure is also missing statistical analysis. Also, how were the cells quantified for granules? What in fiji did you use? Was it manual, was it with a software?

R: We have included the information specifying data analysis in the figure legend and methods. In brief, for each condition and biological replicate, at least six random fields were acquired and a total number of cells ranging from 200 to 350 cells were manually counted in FIJI for total number of cells and granule-positive cells.

We did not include statistical analysis in **Fig 2B and 2C** as these graphs were intended for descriptive quantification of the overall proportion of granule-positive cells across conditions and time points. Statistical analyses were included for other graphs, as suggested.

Figure 4B. It is claimed that there are RLB-like granules observed in the absence of RNAseL when infected with ΔE4. However, there seems to be a drastic difference in these cell lines based on the pictures. There seems to be less RLBs than in the control cells. I would argue that even though the granules didn’t completely disappear, there is something being affected. This would also argue against the conclusion that the mutant virus is inducing a “different type” of aggregate altogether.

R: We agree with the reviewer that the ΔE4-induced granules observed in RNase L KO cells show a reduced number in comparison to granules assembled in parental cells. To measure these differences, we quantified granule number and size in parental and RNase L KO cells. Our analysis showed that ΔE4-induced granules were significantly reduced in number in RNase L KO cells, whereas mean granule area was not significantly changed across different conditions (**S6 Fig C-E**). Similarity in granule size and morphology, together with additional experiments showing recruitment of similar protein markers and polyadenylated RNA, as well as persistence of these granules in the presence of CHX (**Fig 6A-F**), supports the interpretation that these structures retain RLB-like features. However, we hypothesize that differences in granule number could indicate that RNase L contributes to their abundance in infected cells. Therefore, we have revised the manuscript to avoid implying that ΔE4 induces a completely distinct class of granule, and now describe these structures as ΔE4-induced RLB-like granules that persist in the absence of RNase L but are reduced in number (**lines 313-318**).

Figure 4C. What parameters are being used to quantify percent of aggregates? Are # of aggregates per cell considered? Is not considering this affecting the interpretation of the data? The images for the ΔE4 virus clearly show differences in quantity of aggregates per cell. The RNAseL KO cells have very reduced numbers of aggregates per cell compared to the WT and PKR KO. How exactly are these cells being quantified and are the quantifications being done blindly to prevent biases (i.e. counting a SG cell as a RLB cell and viceversa)? This figure is also missing statistics.

R: Percentage of granule-positive cells was measured by acquisition of at least six random fields for each condition and biological replicate, counting between 200 and 350 cells, with quantification based on the identification of G3BP1/PABPC1 granules in the cytoplasm. Granule distinction was based on morphological differences and associated phenotypes, since RNase L-driven RLB assembly accompanies PABPC1 nuclear import. In our initial analysis, we considered only the proportion of granule-positive cells to distinguish between conditions. Data acquired independently and analyzed by statistical tests confirm similar proportion of cells positive for ΔE4-induced RLB-like granules in the three cell lines compared (A549 parental, PKR KO, RNase L KO cells), as reported in **Fig 4D**.

Taking the reviewer’s suggestion into consideration, we also quantified the total number of RLB or RLB-like granules formed in parental or RNase L KO A549 cells in response to poly(I:C) or ΔE4 infection. By comparing the number of granules per cell we have confirmed reduced number of ΔE4-induced aggregates in parental and RNase L KO cells, as described above (**S6 Fig C-E**). The number of cells analyzed, biological replicates, and method of analysis are described in the figure legend and methods. In summary, these results indicate that RNase L is not strictly required for ΔE4-induced G3BP1-positive granule formation. However, because RNase L-deficient cells appear to show reduced granule accumulation, we cannot exclude a quantitative contribution of RNase L to granule formation in infected cells.

Figure 5B and 5C. Why do the violin plots have statistics given that it is only representative of one biological replicate? Taking each individual cell as its own biological replicate would inflate the results and any small difference would be “statistically significant”. I would remove the statistics or redo the experiment two more times, using the means to calculate statistical differences.

R: We have followed the reviewer’s suggestion and quantified *GAPDH* smFISH signal in three biological replicates, with the number of cells and number of biological replicates added to the description in the figure legend. Data were presented as a per cell number (small dots) and mean value (big dots) for each individual replicate. New biological replicates presented the same tendency, with reduced *GAPDH* mRNA signal in parental cells for poly(I:C) and ΔE4 conditions, in agreement with RNase L activity, while *GAPDH* mRNA signal remained constant during poly(I:C) and ΔE4 infection in RNase L KO cells.

Figure 7D. Once again, the images selected to represent the ΔE4 granules are not ideal. Yes, I see the PABPC1 in the nucleus, but I do not see any RLBs in the DMSO or ISRIB treated, suggesting that the double KO does disassemble the “RLB-like” granules. Is presence of PABPC1 in the nucleus sufficient to conclude these are not RLBs or are you using the actual aggregates to confirm their presence/identity?

R: We agree with the reviewer that nuclear PABPC1 relocalization alone is not sufficient to define the assembly of RLB-like granules, with PABPC1 also staining weakly for granules in comparison to G3BP1, FXR1, or other markers tested. Our interpretation that RLB-like granules persist in PKR / RNase L double-KO cells is based on the presence of cytoplasmic granules stained by G3BP1, rather than nuclear PABPC1 alone.

We also agree that the representative ΔE4 images in **Fig 7D** were not ideal for visualizing granules. We have therefore included additional representative fields showing that ΔE4-induced cytoplasmic granules persist in A549 PKR/RNase L double-KO cells (**S7 Fig C**). These images support that the granules are not fully disassembled in the absence of both PKR and RNase L.

PABPC1 staining was used in **Fig 7D** because it distinguishes sodium arsenite-induced SGs from ΔE4-induced RLB-like granules, particularly through PABPC1 nuclear relocalization during ΔE4 infection. However, to avoid overstating that translational arrest is exclusively associated with granule assembly, we have revised the text to clarify that puromycin intensity was quantified in cells positive for stress granules (sodium arsenite), or nuclear PABPC1 and cytoplasmic granules for ΔE4 infection.

**Reviewer #2**

**Part I – Summary**

The manuscript entitled “Differential assembly of RNP granules via activation of distinct dsRNA sensors by adenovirus mutants” presents a comprehensive and well-executed study investigating how different adenovirus mutants (HAdV-C5 WT, ΔE4, and ΔVA) differentially activate dsRNA sensing pathways and drive the formation of RNP granules. The study is technically strong, and the manuscript is clearly written and logically structured. Overall, the work provides valuable insights into virus-host interactions and innate immune sensing. The manuscript is likely suitable for publication in PPath after addressing the points below, which mainly concern clarification, quantification, and strengthening data interpretation.

R: We appreciate the reviewer’s positive assessment of the manuscript and constructive suggestions. We have revised the manuscript to address all comments and suggestions.

**Part II – Major Issues**

1. The Ad5 WT virus was obtained from ATCC, whereas the ΔE4 and ΔVA mutants were sourced from different laboratories. This raises concerns regarding their genetic comparability: Are the mutant viruses derived from the same parental WT backbone? Have the authors verified the integrity of the viral genomes (e.g., by sequencing)? …it is important to ensure that the observed phenotypes are due to the intended deletions rather than unintended mutations elsewhere in the genome. If not experimentally addressed, this limitation should at least be discussed.

R: The Ad5 mutants used in this study, specifically the ΔE4 mutant (dl1004) and the ΔVA mutant (dl-sub720), were obtained from laboratories where these established Ad5 mutants have been maintained and used in prior studies. We agree that these viruses should not be considered a newly generated, sequence-verified model, relative to our Ad5 WT stock.

Although we did not perform whole genome sequencing of the viral stocks used in this study, our observations support that the phenotypes reported here are associated with the intended deletions. First, both mutants reproduced their expected phenotypes, including activation of the PKR pathway during ΔVA infection and the previously reported defects associated with loss of E4 proteins. Second, for ΔE4 infection, previous studies showed that two independently generated E4-deleted viruses, dl1004 and dl366, produced similar dsRNA phenotypes, reducing the likelihood that the phenotype is caused by unintended mutations [5]. Third, the ΔE4-associated phenotype was complemented in W162 cells, which provide the Ad5 E4 region, rescuing functions performed by E4 proteins [7].

We have revised the manuscript to acknowledge this limitation. Since mutant viruses were not generated from the same parental WT virus and were not sequenced in this study, we cannot exclude the possibility that additional sequence differences besides the intended deletions could contribute to some of the phenotypes (**Lines 406-410**).

2.In Figure 1A, only a subset of ΔE4-infected cells appears to show dsRNA accumulation, while many cells do not….: What fraction of infected cells exhibits this phenotype? Quantification of dsRNA-positive cells would strengthen this observation and clarify the extent of the effect!

R: This observation is correct, only a subset of ΔE4-infected cells shows dsRNA accumulation. This observation was previously characterized and published by our group for different ΔE4 mutants, with quantification showing that dsRNA is detected in approximately 40% of cells during ΔE4 infection in A549 cells at 48hpi (See Fig 1E in Price *et al.,* 2022) [5]. This is similar to the proportion of RLB-positive cells during ΔE4 infection, as quantified in **Fig** **2C** and **4D**. We have included mention of this observation in the text (**Lines 219-221**).

3.In Figure 5A, it is unclear whether the displayed WT cells are indeed infected. Please include an infection marker or clarify how infection was verified in these images. Moreover, the data suggest that ΔE4 infection strongly reduces GAPDH mRNA levels. Given the relatively short half-life of GAPDH, it is surprising that GAPDH protein levels remain stable and are used as a loading control in the westerns. Please clarify this apparent discrepancy… consider validating an alternative loading control or discussing protein stability under these conditions!

R: Infections were performed at high MOI, with broad coverage of virus per cell, and infection was confirmed in independent assays by staining for the viral protein DBP, as shown in **Supplementary Fig 2A**. Infected cells also showed cytopathic effect, as evidenced by changes in cell morphology and nuclei enlargement.

Regarding the reduction in mRNA levels, RNase L activation is associated with broad cleavage of cytoplasmic mRNAs, with *GAPDH* transcripts used here as a marker of RNase L-dependent RNA decay. According to our observations, RNase L activation peaks at late time points during ΔE4 infection, between 40h and 48hpi (**Fig 1F**), which also matches detection of dsRNA [5] and granule assembly in about 30% of cells (quantified in **Fig 2A and 2C**). Considering this, we note that reduction in *GAPDH* mRNA levels is expected to occur only in a subset of cells, whereas immunoblotting detects total protein from the entire infected cell population. Besides, protein levels are expected to be less immediately affected by RNase L-mediated transcript cleavage considering the short gap of activation for RNase L activation during ΔE4 infection. Consistent with this, several proteins besides GAPDH, including total PKR, eIF2α, and IRF3, presented comparable expression levels (**Fig 1C, Fig 4A**), suggesting that the observed *GAPDH* mRNA reduction does not reflect a global loss of protein for the time points analyzed.

**Part III – Minor Issues**

1.Line 212: Please verify whether the correct reference is “Fig. 1F”.

R: The correct reference is “**Fig 1C, F**”, as PKR and RNase L kinetics are measured by western blot in **Fig 1C** and bioanalyzer in **Fig 1F**, respectively.

2.It would be helpful to explain why DBP staining was not included in this panel, especially since it is used elsewhere to mark infection.

R: In the panels where DBP is not used, we chose to do co-staining between G3BP1 and PABPC1 (or other protein markers) as both proteins were previously used as markers to distinguish between stress granules and RNase L-dependent bodies.

3.In the infection experiments, how was equal MOI or infectious dose ensured across WT and mutant viruses?

R: Viruses were titered by counting plaque forming units in conventional plaque assays, as described in methods. Ad5 WT virus was titered in HEK293 cells. The ΔVA and ΔE4 mutants were titered in A549 PKR KO and W162 cells, respectively. To ensure similar viral dose across infections, titers for Ad5 WT were also acquired in the respective complementing cell lines and numbers were normalized to the original titer acquired in HEK293 cells. We have included a clearer explanation for this procedure in the methods (**lines 764-766**).

4.In Figure 2A (WT infection), small punctate cytoplasmic structures are visible, and some signal is also observed in the nucleus: Could these represent early or RLBs granules? The authors should clarify or discuss these observations to avoid ambiguity.

R: We have not observed cytoplasmic structures stained by either G3BP1 or PABPC1 during Ad5 WT infection. Additional images staining for G3BP1 and the viral protein DBP were included in the supplementary figures (**S2 Fig A**) where no cytoplasmic granules were seen.

5.The number of analyzed cells is not specified in Figures 2B and 4C… please indicate how many cells (and/or independent experiments) were included in the quantifications.
R: We have included information for the number of cells analyzed and biological replicates in the mentioned figure legends and other figures.

6.Across several IF images, cell sizes appear to differ, while scale bars remain identical. Best example: ΔE4-infected cells in Fig. 6B appear larger. Please check that magnification and scaling are consistent across images.

R: Magnification and scaling are consistent across images. During adenovirus infection, cytopathic effect is observed with cell enlargement, as previously described by Avgousti *et al.*, 2016 and Charman *et al*. 2023 [8,9]. Differences in cell size between uninfected and infected cells are consistent across different IF panels.

7.The manuscript would benefit from explicitly stating why DBP staining was used.
R: DBP is a viral DNA binding protein highly expressed in the early phase, with constant expression across the late phase of infection. It stains for viral replication centers in the nucleus, thus serving as a clear marker of infection. We have stated in the text the use of DBP as a marker for infection in the respective panels in which it was used.

8.I recommend also discussing adenovirus detection via the cGAS/STING/TBK1 DNA sensing pathway, as described by Lam et al., as the canonical reference for adenoviral dsDNA sensing.

R: We appreciate the suggestion and agree that adenovirus can be detected through DNA-sensing pathways, as described by Lam et al. However, our study focuses on RNA-sensing downstream of adenoviral double-stranded RNA formation and VA RNA loss, exploring activation of PKR and OAS3/RNase L, translational arrest, and RNP granule assembly at late stages of infection. In comparison, detection of viral DNA by cGAS/STING is reported to happen at early stages of infection, which is inhibited by E1A, with no clear impact in late infected cells. Since our study does not examine cGAS/STING/TBK1 activation and its contribution to granule formation, focusing on pathways involved with detection of dsRNA, we preferred to not discuss this work.

9.Ensure consistent terminology throughout the manuscript: “WT Ad5” vs. “Ad5 WT”, “Mock” vs. “untreated”, consistent capitalization of labels and terms.

R: We have revised and corrected differences in labeling across conditions.

**Reviewer #3**

**Part I – Summary**

EVALUATIVE STATEMENT

The manuscript entitled “Differential assembly of RNP granules via activation of distinct dsRNA sensors by adenovirus mutants” (PPATHOGENS-D-26-00682) is very well written with exceptionally clear figures and methodology. The discussion is thoughtful and appropriate with insightful suggestions for the existence of novel mechanisms or pathways that target specific classes of mRNA. This report provides new and important information regarding these possibly novel antiviral pathways.

Statistical measures are appropriate and well-described. The supplemental material includes the large dataset associated with proteomics, experimental details supporting conclusions from knock-out cell lines, and experimental replication in other cell lines. This added material is appropriate as supplementary material.

SYNOPSIS
Condensates of ribonucleoprotein may be a by-product of antiviral pathways or an active entity in virus defense. Two classes of such condensates, which are not unique to virus-infected cells, include stress granules and RNase L-dependent bodies. Stress granules play the more general role of protecting cells from stress while RNase L-dependent bodies are the consequence of widespread RNA degradation. RNA degradation is often associated with the antiviral response to double-stranded RNA.

Prior research from the Weitzman group showed that double-stranded RNA is not formed during infection with the wild-type adenovirus despite the presence of many viral transcripts that could hybridize with each other. This finding was repeated here using a novel reagent to detect double-stranded RNA. As previously reported, double-stranded RNA accumulates after infection with a splicing-defective adenovirus mutant deleted of the E4 region. This virus activates both PKR and RNase L pathways resulting in condensates resembling RNase L-dependent bodies. Another mutant virus that fails to express the non-coding viral VA RNA activates PKR in a double-stranded RNA-independent manner and results in the formation of the more general PKR-dependent stress granules.

As a control, these two classes of condensates or granules were formed by treating cells with sodium arsenite or poly(IC). Since both RNase L-associated bodies and stress granules contain the scaffolding protein G3BP1, components of the chemically induced granules were compared to the virally induced granules by proximity labeling with G3BP1 fused to a reagent (APEX2) that biotinylates proteins in the vicinity of the fusion protein.

This proximity labeling method was used in a modified HEK-293 cell line. Additional cell lines were studied by immunolocalization of key cellular and viral proteins and in-situ localization of poly(A) RNA. By analyzing cells unable to express PKR and RNase L, the assembly of these cytoplasmic condensates and consequent inhibition of protein synthesis was found to be independent of both PKR and RNase L. This is the significant contribution of this manuscript, which includes the discovery that OAS3 (and not the more frequently identified OAS1 or OAS2) is the cellular product that collaborates with RNase L.

These findings imply the existence of novel or alternative means of forming RNP granules in response to the accumulation of double-stranded RNA or other facets of a viral infection. This points to the existence of novel antiviral pathways that are targeted by yet-defined adenoviral mechanisms to defeat these pathways. One implication is that these undefined sensors may act in the nucleus to respond to double-stranded RNA leading to cytoplasmic manifestations in the form of bodies resembling RNase L-dependent bodies and translational shutoff.

R: We thank the reviewer for the thorough assessment of our study and appreciation of its significance. We have revised the manuscript to address the comments and broadened the discussion to consider additional non-canonical pathways contributing to translational repression and granule formation during ΔE4 infection.

**Part II – Major Issues**

No major issues

**Part III – Minor Issues**

1. The expression “differentially modulates dsRNA sensors” in the Abstract is vague. Perhaps this could be replaced with a more explicit statement of the observation although I am at a loss as to what to suggest.

R: Following the suggestion, we modified the sentence in the abstract to “adenovirus mutants engage distinct RNA-sensing pathways” to make it less vague.

2. Can a statistical metric be added to quantify the similarity (or differences) in proteins associated with virus-induced and dsRNA-induced RNase L-associated bodies? These were noted to be similar but shared fewer proteins in common than were shared between virally and chemically induced stress granules.

R: For all the proteins tested in our study, we observed similar recruitment of RBPs to both ΔE4- and poly(I:C)-induced RLB or RLB-like bodies. We did observe that some proteins were recruited to SGs, but were absent from granules induced by both ΔE4 and poly(I:C), in accordance with previous observations [11]. In our report, we have focused on characterizing whether cells become granule-positive or not and how differences between SGs and RLBs are reproduced across chemical and viral stress. We agree that further studies quantifying differences in RBP recruitment between chemical and viral-induced granule may offer further detail into the difference between these systems.

3. It is curious that RNA degradation was only evident after 48 h of infection. This seems like a rather late time after infection, especially for a cell line that supports the rapid progression of the adenovirus infection cycle. Why might this be so and could this point to a much later activity in the virus life cycle such as virion assembly or nuclear breakdown?

R: A549 cells are in fact permissive for Ad5 WT infection. However, mutants lacking the E4 regions are highly attenuated, as E4 proteins are necessary to prevent the targeting of viral genomes and transcripts by cellular proteins[1–4]. For this reason, infection has a slower progression for this mutant, with production of viral late transcripts and, consequently, viral double-stranded RNA, only detected at late time points, in agreement with previous observations [5]and activation of PKR (**Fig 1C**) and RNase L (**Fig 1F**).

4. Similarly, the puromycin pulse-labeling method showed that the E4-mutant virus inhibited translation but only at very late times of infection. This effect was independent of PKR, RNase L and eIF2α phosphorylation. Again, why might this be so and what activities so late after infection could be responsible for this effect? Is there any chance that this is simply a “leaky” phenotype for the E4-mutant?

R: Translation inhibition during ΔE4 infection in cells lacking both PKR and RNase L was observed in cells showing granule assembly or nuclear import of PABPC1. While the full mechanism describing this phenotype is not characterized, we currently hypothesize this effect could be dependent on stress driven by nuclear dsRNA accumulation. Alternatively, because PABPC1 is also imported to nucleus upon transcriptional or splicing arrest [12], it could be that interference with these processes during infection or formation of nuclear dsRNA could be driving granule assembly and translational arrest, which remains to be confirmed. This possibility was included in the discussion (**lines 469-474**).

**References**

1.	Stracker TH, Carson CT, Weitzman MD. Adenovirus oncoproteins inactivate the Mre11-Rad50-NBS1 DNA repair complex. Nature. 2002;418: 348–52. doi:10.1038/nature00863

2.	Araujo FD, Stracker TH, Carson CT, Lee D V, Weitzman MD. Adenovirus type 5 E4orf3 protein targets the Mre11 complex to cytoplasmic aggresomes. J Virol. 2005;79: 11382–91. doi:10.1128/JVI.79.17.11382-11391.2005

3.	Reyes ED, Kulej K, Pancholi NJ, Akhtar LN, Avgousti DC, Kim ET, et al. Identifying host factors associated with DNA replicated during virus infection. Molecular and Cellular Proteomics. 2017;16: 2079–2097. doi:10.1074/mcp.M117.067116

4.	Herrmann C, Dybas JM, Liddle JC, Price AM, Hayer KE, Lauman R, et al. Adenovirus-mediated ubiquitination alters protein-RNA binding and aids viral RNA processing. Nat Microbiol. 2020;5: 1217–1231. doi:10.1038/s41564-020-0750-9

5.	Price AM, Steinbock RT, Di C, Hayer KE, Li Y, Herrmann C, et al. Adenovirus prevents dsRNA formation by promoting efficient splicing of viral RNA. Nucleic Acids Res. 2022;50: 1201–1220. doi:10.1093/nar/gkab896

6.	Weinberg DH, Ketner G. A cell line that supports the growth of a defective early region 4 deletion mutant of human adenovirus type 2. Proc Natl Acad Sci U S A. 1983;80: 5383–6. doi:10.1073/pnas.80.17.5383

7.	Price AM, Steinbock RT, Lauman R, Charman M, Hayer KE, Kumar N, et al. Novel viral splicing events and open reading frames revealed by long-read direct RNA sequencing of adenovirus transcripts. PLoS Pathog. 2022;18: e1010797. doi:10.1371/journal.ppat.1010797

8.	Avgousti DC, Herrmann C, Kulej K, Pancholi NJ, Sekulic N, Petrescu J, et al. A core viral protein binds host nucleosomes to sequester immune danger signals. Nature. 2016;535: 173–7. doi:10.1038/nature18317

9.	Charman M, Grams N, Kumar N, Halko E, Dybas JM, Abbott A, et al. A viral biomolecular condensate coordinates assembly of progeny particles. Nature. 2023;616: 332–338. doi:10.1038/s41586-023-05887-y

10.	Kamel W, Segerman B, Öberg D, Punga T, Akusjärvi G. The adenovirus VA RNA-derived miRNAs are not essential for lytic virus growth in tissue culture cells. Nucleic Acids Res. 2013;41: 4802–4812. doi:10.1093/nar/gkt172

11.	Burke JM, Lester ET, Tauber D, Parker R. RNase L promotes the formation of unique ribonucleoprotein granules distinct from stress granules. J Biol Chem. 2020;295: 1426–1438. doi:10.1074/jbc.RA119.011638

12.	Angel M, Fleshler E, Atrash MK, Kinor N, Benichou JIC, Shav-Tal Y. Nuclear RNA-related processes modulate the assembly of cytoplasmic RNA granules. Nucleic Acids Res. 2024;52: 5356–5375. doi:10.1093/nar/gkae119
