# Supplementary material for: Differential assembly of RNP granules via activation of distinct dsRNA sensors by adenovirus mutants
Source: PLoS Pathog. 2026 Jul 24;22(7):e1014452. doi: 10.1371/journal.ppat.1014452 (PMC13426940; doi:10.1371/journal.ppat.1014452)
Supplement: Attachment — Submitted filename: PPATHOGENS-D-26-00682_Review.docx [file ppat.1014452.s010.docx]

In this manuscript, Steinbock et al demonstrate how different adenovirus (Ad5) mutants induce pathways that lead to different molecular aggregate formation, including PKR-dependent stress granules (SGs) and RNAseL-dependent bodies (RLBs). Although a very thorough study, I struggled to grasp the novelty since most of the data included is confirming the structural properties of the aggregates that have been previously described (mostly by the Parker lab), only this time using Ad5 to induce them. I am also not convinced that the granules induced by the $\Delta$E4 mutant are distinct from RLBs, especially because the images selected to support this claim are not convincing. Additionally, some of the figures seem to be repetitive, demonstrating the same conclusion in different ways. I recommend merging some of these figures or changing the order for a more cohesive story. I also recommend shifting the rhetoric (especially in the discussion) to focus on how these pathways are being inhibited by the WT virus and how the effect of having these aggregates formed in the mutants affect the outcome of the infection. That way, you can focus on the importance for the virus to block these pathways, rather than focusing on phenomena that doesn’t even occur in the WT virus. This shift can also reduce the “me too” feel of the study.

Major comments:

Line 119: It is unclear what the $\Delta$ E4 mutant virus is. What is E4 and why does the virus produce dsRNA in its absence? I recommend adding more background on this mutant to make sense of why it is being used and why it produces dsRNA. This is only briefly mentioned in the results.

Figure 1A. It is interesting that you do not see mitochondrial dsRNA in the WT or $\Delta$VA. Could you provide a reason why this is? Is the fluorescent exposure and background removal for all conditions the same? You also say that the $\Delta$ E4 increases production of dsRNA in the nucleus, but there seems to be a lot in the cytoplasm that might explain the PKR activation.

Figure 1C. Your $\Delta$E4 mutant virus has drastically lower levels of viral proteins. Do these viruses replicate similarly? If not, is it even fair to compare activation of sensors and pathways? I would like to see viral replication kinetic data to confirm these mutant viruses are not highly attenuated compared to the WT.

Figure 2A. Are these aggregates forming specifically in infected cells or bystander cells? Could the induction be occurring in a viral-RNA independent matter? There is evidence that host dsRNA can induce PKR activation (Kim et al 2018, Molecular Cell). I would like to see aggregate + viral protein staining to determine if the pathway induction is dependent on the virus being inside of the cell or is it through a paracrine manner.

Figure 2B. The figure legend should clarify how many cells were counted. 4 out of 10 cells and 80 out of 200 is still 40%, but it doesn’t mean the same in terms of significance. The figure is also missing statistical analysis. Also, how were the cells quantified for granules? What in fiji did you use? Was it manual, was it with a software?

Figure 1C and 4A: WB analysis show extremely different p-PKR intensity levels. Figure 1C shows more p-PKR in the $\Delta$ VA and figure 4A shows drastically more p-PKR in the $\Delta$ E4. Are the cells being infected with the same amount of virus? What explains this discrepancy?

Figure 4B. It is claimed that there are RLB-like granules observed in the absence of RNAseL when infected with $\Delta$ E4. However, there seems to be a drastic difference in these cell lines based on the pictures. There seems to be less RLBs than in the control cells. I would argue that even though the granules didn’t completely disappear, there is something being affected. This would also argue against the conclusion that the mutant virus is inducing a “different type” of aggregate altogether.

Figure 4C. What parameters are being used to quantify percent of aggregates? Are # of aggregates per cell considered? Is not considering this affecting the interpretation of the data? The images for the $\Delta$ E4 virus clearly show differences in quantity of aggregates per cell. The RNAseL KO cells have very reduced numbers of aggregates per cell compared to the WT and PKR KO. How exactly are these cells being quantified and are the quantifications being done blindly to prevent biases (i.e. counting a SG cell as a RLB cell and viceversa)? This figure is also missing statistics.

Figure 5B and 5C. Why do the violin plots have statistics given that it is only representative of one biological replicate? Taking each individual cell as its own biological replicate would inflate the results and any small difference would be “statistically significant”. I would remove the statistics or redo the experiment two more times, using the means to calculate statistical differences.

Figure 6. I am not sure how much is this figure contributing to the argument? Figure 3 has already characterized the aggregates in all conditions, this is just adding the RNAseL KO? I think it would be helpful to put these data closer to figure 3, so that it doesn’t feel like the same point is being made and it is rather a continuation of the point.

Figure 7D. Once again, the images selected to represent the $\Delta$ E4 granules are not ideal. Yes, you see the PABPC1 in the nucleus, but I do not see any RLBs in the DMSO or ISRIB treated, suggesting that the double KO does disassemble the “RLB-like” granules. Is presence of PABPC1 in the nucleus sufficient to conclude these are not RLBs or are you using the actual aggregates to confirm their presence/identity?

General comment: All infections with KO cells should be accompanied with infection kinetics (titers or qPCR) to ensure that the differences observed are not due to differences in viral replication. Are the granules not present because of the protein is KO or because the virus is not replicating at all in these cells? Or is the virus replication 10 times more in these cells? WB data is not very quantitative and not very informative when it comes to comparing “infections levels”.
