# Supplementary material for: Differential assembly of RNP granules via activation of distinct dsRNA sensors by adenovirus mutants
Source: PLoS Pathog. 2026 Jul 24;22(7):e1014452. doi: 10.1371/journal.ppat.1014452 (PMC13426940; doi:10.1371/journal.ppat.1014452)
Supplement: Peer review history

# Peer review: Decision Letter 0

**DOI:** 10.1371/journal.ppat.1014452.r001

**Authors:** Donna Neumann, Eain Murphy

21 Apr 2026

-->PPATHOGENS-D-26-00682

Differential assembly of RNP granules via activation of distinct dsRNA sensors by adenovirus mutants

PLOS Pathogens

Dear Dr. Weitzman,

Thank you for submitting your manuscript to PLOS Pathogens. After careful consideration, we feel that it has merit but does not fully meet PLOS Pathogens's publication criteria as it currently stands. Therefore, we invite you to submit a revised version of the manuscript that addresses the points raised during the review process.

Please submit your revised manuscript by Jun 20 2026 11:59PM. If you will need more time than this to complete your revisions, please reply to this message or contact the journal office at plospathogens@plos.org. When you're ready to submit your revision, log on to https://www.editorialmanager.com/ppathogens/ and select the 'Submissions Needing Revision' folder to locate your manuscript file.

Please include the following items when submitting your revised manuscript:

* A letter that responds to each point raised by the editor and reviewer(s). You should upload this letter as a separate file labeled 'Response to Reviewers'. This file does not need to include responses to any formatting updates and technical items listed in the 'Journal Requirements' section below.

* A marked-up copy of your manuscript that highlights changes made to the original version. You should upload this as a separate file labeled 'Revised Manuscript with Track Changes'.

* An unmarked version of your revised paper without tracked changes. You should upload this as a separate file labeled 'Manuscript'.

If you would like to make changes to your financial disclosure, competing interests statement, or data availability statement, please make these updates within the submission form at the time of resubmission. Guidelines for resubmitting your figure files are available below the reviewer comments at the end of this letter.

We look forward to receiving your revised manuscript.

Kind regards,

Eain A Murphy, Ph.D.

Academic Editor

PLOS Pathogens

Donna Neumann

Section Editor

PLOS Pathogens

Sumita Bhaduri-McIntosh

Editor-in-Chief

PLOS Pathogens

orcid.org/0000-0003-2946-9497

Michael Malim

Editor-in-Chief

PLOS Pathogens

orcid.org/0000-0002-7699-2064

**Additional Editor Comments:**

Dear Matt,

Thank you for your submission to PLoS Pathogens. I do apologize for the extended review timeline as one reviewer was slightly tardy in returning a review. That being said, all three reviewers were highly enthusiastic on the work. All three found the work of high importance and relevant for a journal such as PLoS Pathogens. Some minor experimental concerns were raised which the editorial staff believes are easily addressable. While the decision is Major Revision, I am confident that a modified submission addressing the concerns of the reviewers would be favorably received and most likely would receive a positive outcome without further external review. I look forward to having the revised manuscript reviewed by myself.

Cheers,

Eain Murphy

**Journal Requirements:**

1) Please ensure that the CRediT author contributions listed for every co-author are completed accurately and in full.

At this stage, the following Authors/Authors require contributions: Robert T Steinbock, Orlando B Scudero, Joseph M Dybas, Amber R N Abbott, Katarzyna Kulej, Richard Lauman, Holly Chan, Eva L Agostino, Namrata Kumar, Skyler Briggs, Nicholas A Parenti, Yize Li, James M Burke, Susan R Weiss, Alexander M Price, and Matthew D Weitzman. Please ensure that the full contributions of each author are acknowledged in the "Add/Edit/Remove Authors" section of our submission form.

The list of CRediT author contributions may be found here: https://journals.plos.org/plospathogens/s/authorship#loc-author-contributions

2) Please amend your detailed Financial Disclosure statement. This is published with the article. It must therefore be completed in full sentences and contain the exact wording you wish to be published.

- State the initials, alongside each funding source, of each author to receive each grant. For example: "This work was supported by the National Institutes of Health (####### to AM; ###### to CJ) and the National Science Foundation (###### to AM)."

- State what role the funders took in the study. If the funders had no role in your study, please state: "The funders had no role in study design, data collection and analysis, decision to publish, or preparation of the manuscript.".

If you did not receive any funding for this study, please simply state: u201cThe authors received no specific funding for this work.u201d

**Reviewers' Comments:**

Reviewer's Responses to Questions

**Part I - Summary**

Please use this section to discuss strengths/weaknesses of study, novelty/significance, general execution and scholarship.

Reviewer #1: In this manuscript, Steinbock et al demonstrate how different adenovirus (Ad5) mutants induce pathways that lead to different molecular aggregate formation, including PKR-dependent stress granules (SGs) and RNAseL-dependent bodies (RLBs). Although a very thorough study, I struggled to grasp the novelty since most of the data included is confirming the structural properties of the aggregates that have been previously described (mostly by the Parker lab), only this time using Ad5 to induce them. I am also not convinced that the granules induced by the ΔE4 mutant are distinct from RLBs, especially because the images selected to support this claim are not convincing. Additionally, some of the figures seem to be repetitive, demonstrating the same conclusion in different ways. I recommend merging some of these figures or changing the order for a more cohesive story. I also recommend shifting the rhetoric (especially in the discussion) to focus on how these pathways are being inhibited by the WT virus and how the effect of having these aggregates formed in the mutants affect the outcome of the infection. That way, you can focus on the importance for the virus to block these pathways, rather than focusing on phenomena that doesn’t even occur in the WT virus. This shift can also reduce the “me too” feel of the study.

Reviewer #2: The manuscript entitled “Differential assembly of RNP granules via activation of distinct dsRNA sensors by adenovirus mutants” presents a comprehensive and well-executed study investigating how different adenovirus mutants (HAdV-C5 WT, ΔE4, and ΔVA) differentially activate dsRNA sensing pathways and drive the formation of RNP granules. The study is technically strong, and the manuscript is clearly written and logically structured. Overall, the work provides valuable insights into virus-host interactions and innate immune sensing. The manuscript is likely suitable for publication in PPath after addressing the points below, which mainly concern clarification, quantification, and strengthening data interpretation.

Reviewer #3: EVALUATIVE STATEMENT

The manuscript entitled “Differential assembly of RNP granules via activation of distinct dsRNA sensors by adenovirus mutants” (PPATHOGENS-D-26-00682) is very well written with exceptionally clear figures and methodology. The discussion is thoughtful and appropriate with insightful suggestions for the existence of novel mechanisms or pathways that target specific classes of mRNA. This report provides new and important information regarding these possibly novel antiviral pathways.

Statistical measures are appropriate and well-described. The supplemental material includes the large dataset associated with proteomics, experimental details supporting conclusions from knock-out cell lines, and experimental replication in other cell lines. This added material is appropriate as supplementary material.

SYNOPSIS

Condensates of ribonucleoprotein may be a by-product of antiviral pathways or an active entity in virus defense. Two classes of such condensates, which are not unique to virus-infected cells, include stress granules and RNase L-dependent bodies. Stress granules play the more general role of protecting cells from stress while RNase L-dependent bodies are the consequence of widespread RNA degradation. RNA degradation is often associated with the antiviral response to double-stranded RNA.

Prior research from the Weitzman group showed that double-stranded RNA is not formed during infection with the wild-type adenovirus despite the presence of many viral transcripts that could hybridize with each other. This finding was repeated here using a novel reagent to detect double-stranded RNA. As previously reported, double-stranded RNA accumulates after infection with a splicing-defective adenovirus mutant deleted of the E4 region. This virus activates both PKR and RNase L pathways resulting in condensates resembling RNase L-dependent bodies. Another mutant virus that fails to express the non-coding viral VA RNA activates PKR in a double-stranded RNA-independent manner and results in the formation of the more general PKR-dependent stress granules.

As a control, these two classes of condensates or granules were formed by treating cells with sodium arsenite or poly(IC). Since both RNase L-associated bodies and stress granules contain the scaffolding protein G3BP1, components of the chemically induced granules were compared to the virally induced granules by proximity labeling with G3BP1 fused to a reagent (APEX2) that biotinylates proteins in the vicinity of the fusion protein.

This proximity labeling method was used in a modified HEK-293 cell line. Additional cell lines were studied by immunolocalization of key cellular and viral proteins and in-situ localization of poly(A) RNA. By analyzing cells unable to express PKR and RNase L, the assembly of these cytoplasmic condensates and consequent inhibition of protein synthesis was found to be independent of both PKR and RNase L. This is the significant contribution of this manuscript, which includes the discovery that OAS3 (and not the more frequently identified OAS1 or OAS2) is the cellular product that collaborates with RNase L.

These findings imply the existence of novel or alternative means of forming RNP granules in response to the accumulation of double-stranded RNA or other facets of a viral infection. This points to the existence of novel antiviral pathways that are targeted by yet-defined adenoviral mechanisms to defeat these pathways. One implication is that these undefined sensors may act in the nucleus to respond to double-stranded RNA leading to cytoplasmic manifestations in the form of bodies resembling RNase L-dependent bodies and translational shutoff.

**********

**Part II – Major Issues: Key Experiments Required for Acceptance**

Please use this section to detail the key new experiments or modifications of existing experiments that should be absolutely required to validate study conclusions.

Generally, there should be no more than 3 such required experiments or major modifications for a "Major Revision" recommendation. If more than 3 experiments are necessary to validate the study conclusions, then you are encouraged to recommend "Reject".

Reviewer #1: Figure 1C. Your ΔE4 mutant virus has drastically lower levels of viral proteins. Do these viruses replicate similarly? If not, is it even fair to compare activation of sensors and pathways? I would like to see viral replication kinetic data to confirm these mutant viruses are not highly attenuated compared to the WT.

Figure 2A. Are these aggregates forming specifically in infected cells or bystander cells? Could the induction be occurring in a viral-RNA independent matter? There is evidence that host dsRNA can induce PKR activation (Kim et al 2018, Molecular Cell). I would like to see aggregate + viral protein staining to determine if the pathway induction is dependent on the virus being inside of the cell or is it through a paracrine manner.

General comment: All infections with KO cells should be accompanied with infection kinetics (titers or qPCR) to ensure that the differences observed are not due to differences in viral replication. Are the granules not present because the protein is KO or because the virus is not replicating at all in these cells? Or is the virus replication 10 times more in these cells? WB data is not very quantitative and not very informative when it comes to comparing “infections levels”.

Reviewer #2: 1.The Ad5 WT virus was obtained from ATCC, whereas the ΔE4 and ΔVA mutants were sourced from different laboratories. This raises concerns regarding their genetic comparability: Are the mutant viruses derived from the same parental WT backbone? Have the authors verified the integrity of the viral genomes (e.g., by sequencing)? …it is important to ensure that the observed phenotypes are due to the intended deletions rather than unintended mutations elsewhere in the genome. If not experimentally addressed, this limitation should at least be discussed.

2.In Figure 1A, only a subset of ΔE4-infected cells appears to show dsRNA accumulation, while many cells do not….: What fraction of infected cells exhibits this phenotype? Quantification of dsRNA-positive cells would strengthen this observation and clarify the extent of the effect!

3.In Figure 5A, it is unclear whether the displayed WT cells are indeed infected. Please include an infection marker or clarify how infection was verified in these images. Moreover, the data suggest that ΔE4 infection strongly reduces GAPDH mRNA levels. Given the relatively short half-life of GAPDH, it is surprising that GAPDH protein levels remain stable and are used as a loading control in the westerns. Please clarify this apparent discrepancy… consider validating an alternative loading control or discussing protein stability under these conditions!

Reviewer #3: No major issues

**********

**Part III – Minor Issues: Editorial and Data Presentation Modifications**

Please use this section for editorial suggestions as well as relatively minor modifications of existing data that would enhance clarity.

Reviewer #1: Line 119: It is unclear what the Δ E4 mutant virus is. What is E4 and why does the virus produce dsRNA in its absence? I recommend adding more background on this mutant to make sense of why it is being used and why it produces dsRNA. This is only briefly mentioned in the results.

Figure 2B. The figure legend should clarify how many cells were counted. 4 out of 10 cells and 80 out of 200 is still 40%, but it doesn’t mean the same in terms of significance. The figure is also missing statistical analysis. Also, how were the cells quantified for granules? What in fiji did you use? Was it manual, was it with a software?

Figure 4B. It is claimed that there are RLB-like granules observed in the absence of RNAseL when infected with Δ E4. However, there seems to be a drastic difference in these cell lines based on the pictures. There seems to be less RLBs than in the control cells. I would argue that even though the granules didn’t completely disappear, there is something being affected. This would also argue against the conclusion that the mutant virus is inducing a “different type” of aggregate altogether.

Figure 4C. What parameters are being used to quantify percent of aggregates? Are # of aggregates per cell considered? Is not considering this affecting the interpretation of the data? The images for the Δ E4 virus clearly show differences in quantity of aggregates per cell. The RNAseL KO cells have very reduced numbers of aggregates per cell compared to the WT and PKR KO. How exactly are these cells being quantified and are the quantifications being done blindly to prevent biases (i.e. counting a SG cell as a RLB cell and viceversa)? This figure is also missing statistics.

Figure 5B and 5C. Why do the violin plots have statistics given that it is only representative of one biological replicate? Taking each individual cell as its own biological replicate would inflate the results and any small difference would be “statistically significant”. I would remove the statistics or redo the experiment two more times, using the means to calculate statistical differences.

Figure 7D. Once again, the images selected to represent the Δ E4 granules are not ideal. Yes, I see the PABPC1 in the nucleus, but I do not see any RLBs in the DMSO or ISRIB treated, suggesting that the double KO does disassemble the “RLB-like” granules. Is presence of PABPC1 in the nucleus sufficient to conclude these are not RLBs or are you using the actual aggregates to confirm their presence/identity?

Reviewer #2: 1.Line 212: Please verify whether the correct reference is “Fig. 1F”.

2.It would be helpful to explain why DBP staining was not included in this panel, especially since it is used elsewhere to mark infection.

3.In the infection experiments, how was equal MOI or infectious dose ensured across WT and mutant viruses?

4.In Figure 2A (WT infection), small punctate cytoplasmic structures are visible, and some signal is also observed in the nucleus: Could these represent early or RLBs granules? The authors should clarify or discuss these observations to avoid ambiguity.

5.The number of analyzed cells is not specified in Figures 2B and 4C… please indicate how many cells (and/or independent experiments) were included in the quantifications.

6.Across several IF images, cell sizes appear to differ, while scale bars remain identical. Best example: ΔE4-infected cells in Fig. 6B appear larger. Please check that magnification and scaling are consistent across images.

7.The manuscript would benefit from explicitly stating why DBP staining was used.

8.I recommend also discussing adenovirus detection via the cGAS/STING/TBK1 DNA sensing pathway, as described by Lam et al., as the canonical reference for adenoviral dsDNA sensing.

9.Ensure consistent terminology throughout the manuscript: “WT Ad5” vs. “Ad5 WT”, “Mock” vs. “untreated”, consistent capitalization of labels and terms

Reviewer #3: 1. The expression “differentially modulates dsRNA sensors” in the Abstract is vague. Perhaps this could be replaced with a more explicit statement of the observation although I am at a loss as to what to suggest.

2. Can a statistical metric be added to quantify the similarity (or differences) in proteins associated with virus-induced and dsRNA-induced RNase L-associated bodies? These were noted to be similar but shared fewer proteins in common than were shared between virally and chemically induced stress granules.

3. It is curious that RNA degradation was only evident after 48 h of infection. This seems like a rather late time after infection, especially for a cell line that supports the rapid progression of the adenovirus infection cycle. Why might this be so and could this point to a much later activity in the virus life cycle such as virion assembly or nuclear breakdown?

4. Similarly, the puromycin pulse-labeling method showed that the E4-mutant virus inhibited translation but only at very late times of infection. This effect was independent of PKR, RNase L and eIF2α phosphorylation. Again, why might this be so and what activities so late after infection could be responsible for this effect? Is there any chance that this is simply a “leaky” phenotype for the E4-mutant?

**********

PLOS authors have the option to publish the peer review history of their article (what does this mean?). If published, this will include your full peer review and any attached files.

If you choose “no”, your identity will remain anonymous but your review may still be made public.

**Do you want your identity to be public for this peer review?**  For information about this choice, including consent withdrawal, please see our Privacy Policy.

Reviewer #1: No

Reviewer #2: No

Reviewer #3: No

[NOTE: If reviewer comments were submitted as an attachment file, they will be attached to this email and accessible via the submission site. Please log into your account, locate the manuscript record, and check for the action link "View Attachments". If this link does not appear, there are no attachment files.]

**Figure resubmission:**

-->While revising your submission, we strongly recommend that you use PLOS’s NAAS tool (https://ngplosjournals.pagemajik.ai/artanalysis) to test your figure files. NAAS can convert your figure files to the TIFF file type and meet basic requirements (such as print size, resolution), or provide you with a report on issues that do not meet our requirements and that NAAS cannot fix.

After uploading your figures to PLOS’s NAAS tool - https://ngplosjournals.pagemajik.ai/artanalysis, NAAS will process the files provided and display the results in the "Uploaded Files" section of the page as the processing is complete. If the uploaded figures meet our requirements (or NAAS is able to fix the files to meet our requirements), the figure will be marked as "fixed" above. If NAAS is unable to fix the files, a red "failed" label will appear above. When NAAS has confirmed that the figure files meet our requirements, please download the file via the download option, and include these NAAS processed figure files when submitting your revised manuscript.

**Reproducibility:**

To enhance the reproducibility of your results, we recommend that authors of applicable studies deposit laboratory protocols in protocols.io, where a protocol can be assigned its own identifier (DOI) such that it can be cited independently in the future. Additionally, PLOS ONE offers an option to publish peer-reviewed clinical study protocols. Read more information on sharing protocols at https://plos.org/protocols?utm_medium=editorial-email&utm_source=authorletters&utm_campaign=protocols

Attachment Submitted filename: PPATHOGENS-D-26-00682_Review.docx

# Author comment: Author response to Decision Letter 1

**DOI:** 10.1371/journal.ppat.1014452.r002

1 Jul 2026

Attachment Submitted filename: Response to Reviewers.docx

# Editor report: Decision Letter 1

**DOI:** 10.1371/journal.ppat.1014452.r003

**Authors:** Donna Neumann, Eain Murphy

8 Jul 2026

Dear Dr. Weitzman,

We are pleased to inform you that your manuscript 'Differential assembly of RNP granules via activation of distinct dsRNA sensors by adenovirus mutants' has been provisionally accepted for publication in PLOS Pathogens.

Before your manuscript can be formally accepted you will need to complete some formatting changes, which you will receive in a follow up email. A member of our team will be in touch with a set of requests.

Please note that your manuscript will not be scheduled for publication until you have made the required changes, so a swift response is appreciated.

IMPORTANT: The editorial review process is now complete. PLOS will only permit corrections to spelling, formatting or significant scientific errors from this point onwards. Requests for major changes, or any which affect the scientific understanding of your work, will cause delays to the publication date of your manuscript.

Should you, your institution's press office or the journal office choose to press release your paper, you will automatically be opted out of early publication. We ask that you notify us now if you or your institution is planning to press release the article. All press must be co-ordinated with PLOS.

Thank you again for supporting Open Access publishing; we are looking forward to publishing your work in PLOS Pathogens.

Best regards,

Eain A Murphy, Ph.D.

Academic Editor

PLOS Pathogens

Donna Neumann

Section Editor

PLOS Pathogens

Sumita Bhaduri-McIntosh

Editor-in-Chief

PLOS Pathogens

orcid.org/0000-0003-2946-9497

Michael Malim

Editor-in-Chief

PLOS Pathogens

orcid.org/0000-0002-7699-2064

***********************************************************

Matt,

I do have to say that after following your scientific trajectory and accomplishments over the last couple of decades, I am pleasantly surprised at the quality of work in this revised manuscript. With a full understanding that a majority of the heavy lifting with the additional experimentation and text revision was most likely performed by your collaborators, all due credit should be afforded to them. It is the opinion of the PLoS Pathogens Editorial Board that this manuscript has made it over the line in terms of now being acceptable for publication without any additional external review. You may want to thank your colleagues.

Cheers,

Eain

Reviewer Comments (if any, and for reference):

# Editor report: Acceptance letter

**DOI:** 10.1371/journal.ppat.1014452.r004

**Authors:** Donna Neumann, Eain Murphy

Dear Dr. Weitzman,

We are delighted to inform you that your manuscript, "Differential assembly of RNP granules via activation of distinct dsRNA sensors by adenovirus mutants," has been formally accepted for publication in PLOS Pathogens.

We have now passed your article onto the PLOS Production Department who will complete the rest of the pre-publication process. All authors will receive a confirmation email upon publication.

The corresponding author will soon be receiving a typeset proof for review, to ensure errors have not been introduced during production. Please review the PDF proof of your manuscript carefully, as this is the last chance to correct any scientific or type-setting errors. Please note that major changes, or those which affect the scientific understanding of the work, will likely cause delays to the publication date of your manuscript. Note: Proofs for Front Matter articles (Pearls, Reviews, Opinions, etc...) are generated on a different schedule and may not be made available as quickly.

Soon after your final files are uploaded, the early version of your manuscript, if you opted to have an early version of your article, will be published online. The date of the early version will be your article's publication date. The final article will be published to the same URL, and all versions of the paper will be accessible to readers.

For Research Articles, you will receive an invoice from PLOS for your publication fee after your manuscript has reached the completed accept phase. If you receive an email requesting payment before acceptance or for any other service, this may be a phishing scheme. Learn how to identify phishing emails and protect your accounts at https://explore.plos.org/phishing.

Thank you again for supporting open-access publishing; we are looking forward to publishing your work in PLOS Pathogens.

Best regards,

Sumita Bhaduri-McIntosh

Editor-in-Chief

PLOS Pathogens

orcid.org/0000-0003-2946-9497

Michael Malim

Editor-in-Chief

PLOS Pathogens

orcid.org/0000-0002-7699-2064
